# Characterization of spontaneous gross and histologic findings in free-ranging Egyptian rousettes (*Rousettus aegyptiacus*) from Uganda with a review of reported pathogens

**DOI:** 10.1186/s13567-026-01785-z

**Published:** 2026-08-27

**Authors:** Jessica A. Elbert, Brian R. Amman, Tara K. Sealy, Patrick Atimnedi, Katy A. Martin, Jonathan S. Towner, Elizabeth W. Howerth

**Affiliations:** 1 Department of Pathology, University of Georgia College of Veterinary Medicine, Athens, GA 30602 USA; 2 Viral Special Pathogens Branch, Division of High-Consequence Pathogens and Pathology, Centers for Disease Control and Prevention, Atlanta, GA 30333 USA; 3 Uganda Wildlife Authority, Kampala, Uganda; 4 Department of Veterinary Pathology, Iowa State University College of Veterinary Medicine, Ames, IA 50011 USA

**Keywords:** Egyptian rousette, *Rousettus aegyptiacus*, histology, pathology, natural reservoir

## Abstract

The emergence of significant viral pathogens and their escalating threats to human and animal health have intensified scientific interest in bats as disease vectors and natural reservoir hosts. Among chiropteran species, the Egyptian rousette (*Rousettus aegyptiacus*) assumes particular epidemiological significance as the established natural reservoir for orthomarburgviruses, encompassing the two recognized members Marburg virus and Ravn virus. While contemporary research predominantly emphasizes the detection and characterization of specific zoonotic agents within these populations, fundamental knowledge regarding their baseline pathology and the comprehensive impacts of disease on morbidity remains limited. Pathological investigations of free-ranging bat populations face inherent methodological constraints, frequently hampered by insufficient sample sizes or selection bias toward compromised specimens (e.g., moribund or deceased animals). This study addresses these limitations through comprehensive post-mortem examination and histologic evaluation of 69 free-ranging Egyptian rousettes from Uganda. These specimens were procured during systematic efforts to identify filoviral reservoir hosts, thereby providing an unprecedented opportunity to investigate spontaneous disease processes within an ostensibly healthy, representative population. Gross pathological examination identified lesions in 85.5% (59/69) of specimens, including scarring and fibrosis of the plagiopatagium and dactylopatagium (85.5%; 59/69), focal fibrotic nodules (17.4%; 12/69), and lacerations (17.4%; 12/69) within wing membranes. Histopathological analysis demonstrated the universal presence of microscopic alterations (100%; 69/69), with a cumulative total of 794 discrete lesions documented across 28 organ systems. The liver emerged as the most frequently affected organ, along with multiorgan perivascular cellular aggregates (both 81.2%; 56/69). This investigation represents the first comprehensive characterization of naturally occurring pathological processes in free-ranging Egyptian rousettes from Uganda. Elucidating the intrinsic factors that modulate reservoir species health is fundamental to understanding zoonotic disease spillover dynamics and developing evidence-based strategies for mitigating future outbreak risks in human and susceptible animal populations.

**Supplementary Information:**

The online version contains supplementary material available at https://doi.org/10.1186/s13567-026-01785-z.

## Introduction

Bats (Order Chiroptera, etymologically derived from “hand wing”) represent one of the most taxonomically diverse mammalian orders, comprising 1500 recognized species as of August 2025 that constitute more than 20% of global mammalian biodiversity [1, 2]. Second only to rodents in species diversity, chiropterans have achieved near-global distribution, inhabiting all continents except Antarctica, and exhibit extraordinary ecological, anatomical, and physiological diversity that is virtually unparalleled within the animal kingdom [1, 3]. Their unique adaptations, including powered flight, echolocation (in most species), and exceptional longevity relative to body size, enable them to fulfill critical ecological roles as pollinators, seed dispersers, and arthropod population regulators [4, 5]. Beyond their fundamental ecological significance, bats have garnered increasing recognition for their relevance to public and animal health as natural reservoir hosts for a wide array of zoonotic viruses [6].

Large-scale surveillance investigations of free-ranging chiropteran populations across diverse biogeographical regions have consistently documented elevated prevalences of mild, frequently subclinical histopathological lesions, reflecting both pathogen exposure histories and distinctive immunobiological characteristics. In Germany, bacterial pathogens were frequently associated with inflammatory lesions, while traumatic injuries, particularly those attributed to domestic cat predation, accounted for nearly half of non-disease-related mortalities [7]. Parallel investigations conducted in Turin Province, Italy, identified trauma and predation as predominant mortality factors in vespertilionid and molossid species, with characteristic histological findings encompassing lymphoplasmacytic pneumonia, dermatitis, hepatic steatosis, hepatitis, and splenic lymphoid depletion [8]. Across both yinpterochiropteran (e.g., *Rousettus* spp., *Pteropus* spp.) and yangochiropteran (e.g., *Myotis* spp., *Tadarida* spp.) lineages, commonly documented lesions include lymphoid hyperplasia, nonsuppurative hepatitis, interstitial nephritis, and lymphohistiocytic pneumonia, frequently manifesting in the absence of overt clinical disease or identifiable pathogenic agents [6, 9, 10]. These pathological changes may reflect chronic viral persistence or environmental stressor responses and are often contextualized within frameworks of reservoir competence for viruses including Marburg virus, Nipah virus, rabies virus, and coronaviruses. A notable pathological exception is white-nose syndrome in North American bat populations, caused by the fungal pathogen *Pseudogymnoascus destructans*, which induces severe histological lesions and has precipitated catastrophic population declines; recent phylogenetic evidence suggests the fungus originated in Western Ukraine [11–13]. Collectively, histopathological findings from surveillance studies underscore bats’ remarkable tolerance of viral infectious agents and their complex, often species-specific interactions with microbial communities of both ecological and zoonotic significance. It has been hypothesized that chiropterans may have evolved enhanced antiviral defense mechanisms, potentially through mitochondrial adaptations associated with flight metabolism, at the evolutionary cost of diminished capacity to eliminate bacterial, fungal, and parasitic infections [6].

The Egyptian rousette (ERB; *Rousettus aegyptiacus*) represents a pteropodid species with extensive geographical distribution across Africa, western Asia, the Mediterranean basin, and the Indian subcontinent [14]. These predominantly cave-dwelling, highly gregarious animals form densely aggregated roosts that may exceed 100 000 individuals [14]. ERBs are of critical epidemiological importance as the established natural reservoir host for both Marburg virus (MARV) and Ravn virus (RAVV), as demonstrated through numerous longitudinal ecological investigations [9, 15–19]. MARV and RAVV, phylogenetically related to Ebola virus, constitute the only recognized members of the species *Orthomarburgvirus marburgense* (family *Filoviridae*, genus *Orthomarburgvirus*) and serve as the etiological agents of Marburg virus disease (MVD), a severe viral hemorrhagic fever typically emerging in sub-Saharan Africa, characterized by human-to-human transmission and case fatality ratios reaching 90% [20]. In stark contrast to the severe, frequently fatal disease observed in humans infected with MARV and RAVV, ERBs remain clinically healthy following experimental inoculation, developing low-level viremia with widespread, multiorgan viral dissemination detectable through molecular diagnostics [16, 21].

The majority of research in free-ranging ERB populations has concentrated on ecological and epidemiological investigations of their role as a natural reservoir for orthomarburgviruses [9, 15, 17–19, 22, 23]. ERBs additionally serve as putative reservoirs for Sosuga virus (SOSV; family *Paramyxoviridae*), and confirmed vertebrate reservoirs for Kasokero virus (KASV; family *Orthonairoviridae*) [24–28]. Recent experimental investigations examining viral coinfection dynamics in ERBs demonstrated that SOSV + MARV and KASV + MARV coinfections differentially modulate MARV shedding patterns and anti-MARV IgG responses, implicating coinfection dynamics as critical determinants of bat-to-bat transmission and spillover potential [29]. Numerous pathogens spanning viral, bacterial, and fungal taxonomic groups, in addition to diverse parasitic species, have been identified in free-ranging ERB populations through serological and molecular approaches. The predominance of reported viral pathogens may reflect research bias toward investigating viral families with established zoonotic potential, or alternatively, may indicate ERBs’ putatively enhanced capacity to tolerate viral infection and replication compared with other mammalian species [30–36]. Previously documented pathogens and parasitic species identified in both free-ranging and managed care ERB populations are discussed below and comprehensively catalogued in Additional file 1.

### Etiologies previously reported in free-ranging Egyptian rousettes

#### Viral pathogens

RNA viruses are of critical importance as high-consequence zoonotic pathogens that originate in wildlife reservoirs. Comprising approximately 94% of zoonotic viruses [37], numerous ecological and biological factors contribute to RNA viruses’ success as pandemic pathogens, including elevated mutation rates and frequency of recombination [38, 39]. Beyond MARV and RAVV [15, 17, 23, 40–44], extensive RNA viral diversity has been documented in free-ranging ERBs through serological and molecular diagnostic approaches. These viruses encompass members of *Coronaviridae* (e.g., Coronavirus sp.) [45–48], *Flaviviridae* (e.g., West Nile virus, Yellow fever virus, Zika virus) [49, 50], *Orthomyxoviridae* (e.g., Influenza A virus) [51]*, Paramyxoviridae* (e.g. Henipavirus, Rubulavirus, Sosuga virus) [26, 27, 52, 53], and *Rhabdoviridae* (e.g., Lagos bat virus) [54–56]. DNA viruses, including members of *Adenoviridae* [57], *Herpesviridae* [58–60], and *Poxviridae* [61, 62], have been additionally documented in free-ranging ERBs, with poxviruses demonstrating associations with morbidity and mortality in Israeli populations and confirmed zoonotic potential [63].

#### Bacterial and fungal pathogens

Reports of bacterial pathogens in free-ranging ERBs remain limited, with even fewer documented cases of clinical illness associated with bacterial infection. ERBs exhibit low colonization rates of *Staphylococcus aureus* and *S. schweitzeri*, phylogenetic analyses suggesting extensive geographical dispersal of *S. schweitzeri* among wildlife hosts [64]. Hemotropic mycoplasmas have been identified in two ERBs in Nigeria [65], while *Leptospira* sp. were identified in South African populations [66]. *Borrelia* sp. were identified in 59 ERBs from a cave epidemiologically linked to human relapsing fever cases following soft tick exposure [67]. *Escherichia coli* was documented in two ERBs from the Republic of Congo [68], and *Bartonella* sp. have been identified in ERBs from Kenya [69, 70], Zambia [71], and South Africa [70, 72]. The only reports of bacterial-associated clinical disease in free-ranging ERBs occur in Israel, where primarily juveniles and young adults are seasonally afflicted with abscessation attributed to *S. aureus* [73]. To our knowledge, no reports of fungal identification or disease exist for free-ranging ERB populations.

#### Parasites

Current knowledge of ectoparasitic fauna in ERBs encompasses diverse arthropod taxa, including bat flies (Nycteribiidae), soft ticks (Argasidae), mites, and fleas, with more than 50 unique species documented throughout the ERB geographical range (see Additional file 1). These ectoparasites serve potential roles in pathogen transmission dynamics; notably, *Eucampsipoda aegyptia*, a host-specific nycteribiid bat fly, has been documented in at least ten countries within the ERB northern range [73–77], with 36.9% [76] to 43.9% [78] polymerase chain reaction (PCR) positivity for *Bartonella* sp. in *E. aegyptia* specimens collected from ERBs across seven countries. Furthermore, the argasid *Ornithodoros (Reticulinasus) faini* functions as a tick vector in the enzootic transmission cycle of Kasokero virus with ERBs [28, 79]. While no evidence supports *O. faini* involvement in enzootic orthomarburgvirus maintenance within ERB populations [80], additional research is required to investigate other ERB ectoparasites and their potential contributions to zoonotic disease dynamics. Experimentally infected *Thaumapsylla breviceps breviceps* fleas inoculated intracelomically with MARV lacked vectorial capacity for biological MARV transmission to ERBs [81], however, ectoparasite roles in mechanical transmission of MARV or other pathogens has yet to be investigated.

Current understanding of endoparasitic fauna in ERBs is limited, with reports primarily documenting protozoal species within the phylum Apicomplexa. *Eimeria rousetti* [82] and the novel pathogen *Gregarina rousetti* n. sp. [83] have been documented in Egyptian ERB populations, as well as numerous *Plasmodium* sp. identified in ERBs in Nigeria [84], Ghana [85], and Congo [86], as well as in Guinea, Liberia, and the Ivory Coast [87]. *Trypanosoma* sp. have been reported in Gabonese [88] and South African [89] populations, with a single documentation of the filarial nematode *Diptalonema* [*sic*] *vitae* in Egyptian ERBs [90]. Pentastomids, crustacean arthropods commonly termed “tongue worms", have been reported on the liver and spleen of ERBs in Uganda [9]. Future endoparasite research in ERBs represents a compelling and underexplored avenue of study, offering significant potential to enhance our understanding of parasite–host dynamics and their implications for both chiropteran health and zoonotic disease transmission.

### Infectious and noninfectious diseases in managed care Egyptian rousettes

#### Iron overload

Reports of clinical disease and pathogen detection in managed care ERBs are limited, with published literature predominantly focusing on iron overload disease. Iron overload disease represents the leading cause of morbidity and mortality in managed care ERBs and in other pteropid species, potentially attributable to increased dietary iron content or environmental exposure in managed care settings [91–99]. ERBs with iron overload disease accumulate iron most prominently in the liver, with additional deposition documented in pancreatic, pulmonary, renal, skeletal muscle, splenic, intestinal, adrenal cortical, vesical, neural, dermal, and reproductive tissues [97]. Recent histopathological comparisons of hepatic iron overload between managed care ERB populations (zoological and research colonies) and free-ranging ERBs identified negative associations between MARV infection status in free-ranging populations and hemosiderosis, suggesting possible hepatoprotective mechanisms through hepcidin upregulation during MARV infection [98]. ERBs demonstrate limited capacity for hepcidin expression upregulation, potentially predisposing to iron overload under dietary iron excess [95]; MARV-infection-associated hepcidin upregulation may counteract this predisposition, though further investigation is required to validate this hypothesis.

#### Infectious pathogens

Reports of viral, bacterial, and fungal infections in managed care ERBs remain sparse. Two ERBs succumbed to European bat lyssavirus 1 following importation into Denmark from a Dutch zoo [55, 100]. Subsequent depopulation of all Danish-imported bats and the original Dutch colony found clinically silent European bat lyssavirus 1 infection in 11.9% and 13% of the ERBs, respectively [55, 56, 100]. Two novel *Betaherpesvirinae* and *Gammaherpesvirinae* subfamily members were detected via nested PCR in pooled hepatic, pulmonary, and small intestinal samples from two deceased ERBs in Hungary [59], while two additional novel *Betaherpesvirinae* members were identified in oropharyngeal samples from clinically healthy ERBs in Spain [58]. In Israel, a novel poxvirus (Israeli *Rousettus aegyptiacus* pox virus; IsrRAPXV) was isolated from managed care ERBs in 2014 [61] and 2019 [62]. One adult female developed clinical but nonfatal poxviral disease with multifocal vesicular and nodular wing membrane lesions [61]; 5 years later, five juvenile ERBs developed fatal IsrRAPXV infection with characteristic pox-like lesions on the ventral abdomen, wing membranes, and tongue [62]. A novel papillomavirus, subsequently designated Rousettus aegyptiacus papillomavirus type 1, was isolated from an ERB from a US-based bat conservation organization presenting with multiple cutaneous papillomas and basosquamous carcinoma in the lateral canthus of the left eye and multifocally throughout left wing membranes [101, 102]. A total of 15 *Bifidobacterium* sp. were identified in environmental fecal swabs from ERBs in a zoological park in Verona, Italy [103]. *Kluyvera ascorbata* was isolated from blood of a deceased ERB in Korea, a pathogen previously documented in variable human clinical infections and subclinically infected Madagascan lemurs [104]. Additionally, yersinosis attributed to *Yersinia pseudotuberculosis* has been reported in three closed ERB managed care colonies, resulting in ~10% mortality [105], 19.7% mortality [106] or complete depopulation [107]. Reports of fungal pathogens are scarce, with a single study detecting *Pneumocystis* sp. in 35.3% (6/17) of ERBs from two managed care colonies in France via nested PCR analysis of pulmonary tissue [108]. A single report documents fatal microsporidiosis attributed to *Encephalitozoon hellem* in a managed care ERB, possibly resulting from mechanical spore transfer from an adjacent aviary [109].

#### Neoplasia

A diverse array of neoplasms have been documented, including adenomas [97, 110], carcinomas [97], sarcomas [97, 111, 112], and lymphomas [113], affecting pulmonary [97, 110, 114], pancreatic [110], hepatic [97, 115], renal [97], vesical [97], and cutaneous tissues [113]. Etiological factors have been proposed in select cases, including microchip-associated leiomyosarcoma [116] and papillomavirus infection linked to a basosquamous carcinoma in wing membranes [101, 102]. Iron overload has been implicated in the pathogenesis of hepatocellular carcinomas; retrospective analysis demonstrated significantly higher hepatocellular carcinoma prevalence in ERBs with hemochromatosis compared with those with hemosiderosis [97]. Additional documented conditions in managed care ERB populations include pneumonia [97, 117], membranous glomerulopathy [97], renal tubular necrosis [97], chronic interstitial nephritis [97], hepatic failure [97], myocardial degeneration and/or fibrosis [97], bacterial sepsis (undisclosed etiology) [97], gallbladder rupture [97], thymic lymphoid depletion [117], hepatocellular degeneration [117], multicentric hyperostosis associated with fluorosis [118], and “skin disease” (results anonymized) in 22% of managed care ERBs from two zoos [119]. Iatrogenic mortality attributed to pulmonary embolism of gelatin hemostatic sponge has been additionally documented in two ERBs [120].

The ERB has garnered increasing scientific interest due to its position at the intersection of public health, ecology, and unique aspects of mammalian biology and immunology, positioning it as a potential model organism for comparative mammalian investigations. While the extensive pathogen diversity identified in ERBs provides valuable insights into infectious pressures confronting this species, these data alone do not provide comprehensive understanding of tissue-level health and disease in free-ranging populations. To date, no systematic histological evaluation of free-ranging ERBs has been conducted; consequently, our understanding of their baseline pathological profiles remains fundamentally limited. Here, we present the first comprehensive assessment of spontaneous gross and histological findings in free-ranging ERBs, establishing a foundational reference that advances understanding of this critical reservoir species as a complete biological entity and supports future investigations into host health, disease ecology, and comparative pathology.

## Materials and methods

A total of 69 Egyptian rousette carcasses were collected in 2008–2009 as part of investigative efforts conducted by the Centers for Disease Control and Prevention (CDC) to identify the orthomarburgvirus natural reservoir host. The study population comprised 49 juveniles (71%) and 20 adults (29%), with a sex distribution of 35 males (50.7%) and 34 females (49.3%). Age determination was performed using previously validated forearm length metrics (juvenile ≤ 89 mm; adult > 89 mm) [9, 121]. All animals were euthanized within 1–3 h of capture.

As previously described, all capturing, processing, and procedures were performed in accordance with an institutionally approved animal care and use protocol [9, 15]. Bat collections were completed with Uganda Wildlife Authority approval and following the American Veterinary Medical Association guidelines on euthanasia and the National Research Council recommendations for the care and use of laboratory animals [9]. Whole blood and splenic tissues were collected at capture for Marburg virus quantitative reverse transcription polymerase chain reaction (RT-qPCR) (negative = 41; positive = 27; unknown = 1). RT-qPCR was completed as previously described [15]. Abdominal ± thoracic cavities were exposed to facilitate 10% neutral buffered formalin fixation, with carcasses fixed as intact specimens. Specimens were subsequently transferred to 70% ethanol following fixation; a definitive timepoint for this transfer is unknown.

In 2023, comprehensive necropsies were performed on each specimen and all macroscopic findings recorded. Representative sections from complete tissue sets (including but not limited to heart, trachea, lungs, lymph nodes, liver, kidney, adrenal gland, tongue, gingiva, esophagus, stomach, pancreas, small and large intestines, haired skin, plagiopatagium, salivary gland, adipose, thymus, thyroid, bone marrow, ear canal, eyes, nasal turbinates, peripheral nerves, skeletal muscle, male and female reproductive organs, urinary bladder, and brain) were processed and embedded in liquid paraffin. Sections were cut at 4 μm thickness, mounted on glass slides, and stained with hematoxylin and eosin (HE). Additional staining methodologies were employed on the basis of microscopic findings, i.e., bacterial detection (Gram staining), fungal identification (Grocott–Gömöri’s methenamine silver staining), iron demonstration (Perls’ Prussian blue stain), and connective tissue and collagen visualization (Masson’s trichrome staining).

## Results

### Post-mortem findings

**Figure 1:**
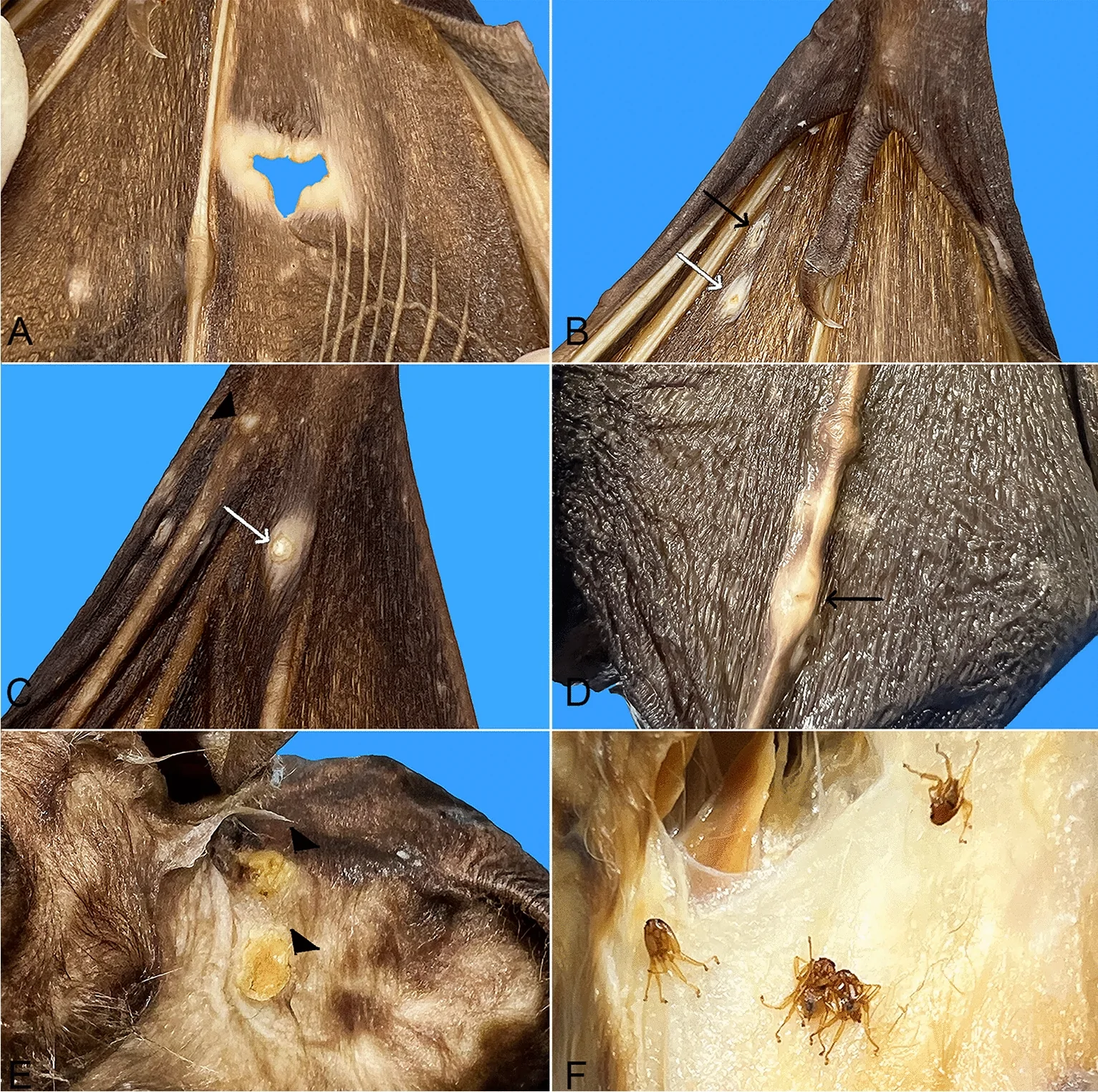
**Macroscopic findings in free-ranging Egyptian rousettes *(Rousettus aegyptiacus)* from Uganda**. **A** Laceration with circumferential fibrosis in the plagiopatagium of a juvenile male. **B** Small laceration (thin black arrow) and fibrotic parasitic nodule (thin white arrow) in the dactylopatagium of a juvenile female. **C** Fibrotic parasitic nodule (thin white arrow) and multifocal dermal fibrosis (black arrowhead) in the dactylopatagium of a juvenile male. **D** Callused mid-diaphyseal fracture (thin black arrow) of the first phalanx of the fifth digit in an adult male. **E** Multifocal ulcerative dermatitis (black arrowheads) in the pericervical region of an adult male. **F** Four Nycteribiidae bat flies in the axillary region of an adult female. The upper limb and humerus are dorsally reflected.

Gross pathological examination revealed lesions in 85.5% (59/69) of examined ERBs, ranging from mild to moderate severity. The predominant findings included scarring and fibrosis (85.5%; 59/69) (Figure 1A), variably sized lacerations (17.4%; 12/69) (Figure 1A, B), and focal fibrotic nodules (17.4%; 12/69) (Figure 1B, C) within the plagiopatagium and dactylopatagium. These lesions are consistent with traumatic injuries commonly documented in free-ranging bat populations, which, depending on the severity of the lesion, can heal rapidly without significant morbidity [122]. Additional gross findings encompassed periosteal hemorrhage (4.3%; 3/69), osseous proliferation (4.3%; 3/69), focal dermal erosion (2.9%; 2/69), healed/callused mid-diaphyseal phalangeal fracture (fifth digit, first phalanx) (1.4%; 1/69) (Figure 1D), subcutaneous hemorrhage (1.4%; 1/69), and multifocal ulcerative dermatitis (1.4%; 1/69) (Figure 1E). One specimen was missing its right eye at capture (1.4%; 1/69), while two females were gravid (2.9%; 2/69). Numerous Nycteribiidae bat flies were observed both free-floating in fixative and adherent to carcasses (Figure 1F). Due to undocumented fixative transfers and carcass manipulations since initial collection circa 2008, precise enumeration was not performed, as counts would likely not represent authentic in vivo ectoparasitic burdens.

### Histopathological findings

Microscopic examination revealed histological alterations in all specimens (100%; 69/69), with 794 discrete lesions documented across 28 organ systems; comprehensive findings are summarized in an additional file (see Additional file 2). The liver emerged as the most frequently affected organ, along with multiorgan perivascular cellular aggregates (both 81.2%; 56/69), the latter categorized as “vascular” lesions for analytical purposes. Notably, no pathological changes were of sufficient severity to have constituted a mortality risk had the animals not been collected for filoviral surveillance purposes.

### Hepatic pathology

**Figure 2:**
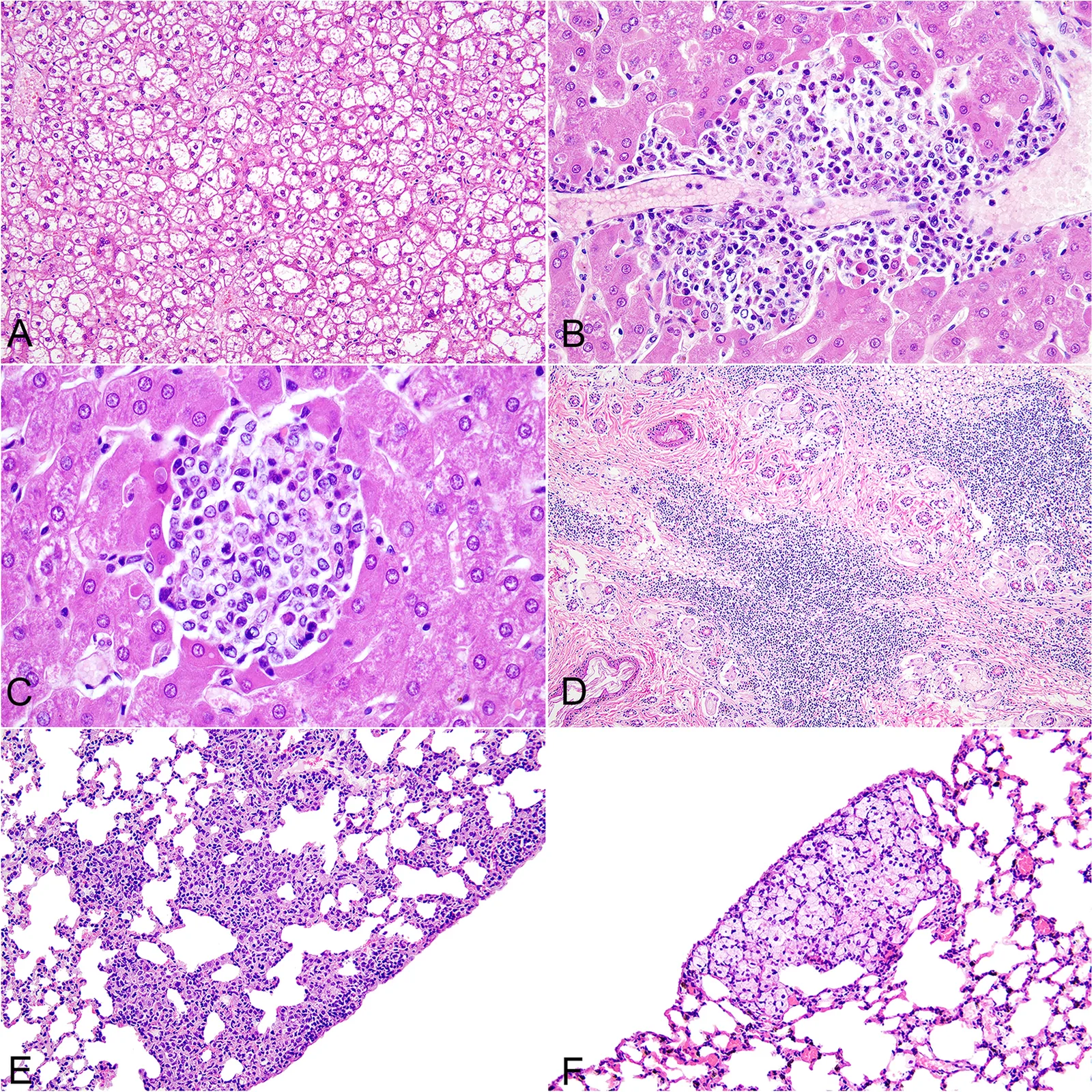
**Histopathologic findings in the liver, dermis, and lung in Egyptian rousettes** (***Rousettus aegyptiacus***). **A** Vacuolar degeneration, glycogen type, in the liver of an adult female. 20×, HE. **B** Perivascular inflammatory focus composed of lymphocytes, plasma cells, macrophages, and few neutrophils, with hepatocellular necrosis, fibrin, and scant hemorrhage in the liver of a juvenile female. This ERB was MARV-RT-qPCR positive (spleen) at capture. 40×, HE. **C** Inflammatory focus composed of lymphocytes, plasma cells, and macrophages in the liver of a juvenile female. This ERB was MARV-RT-qPCR positive (spleen) at capture. 60×, HE. **D** High numbers of lymphocytes, plasma cells, macrophages, and fewer neutrophils dissect through superficial and deep dermal layers of the pelvis in a juvenile female. 10×, HE. **E** Interstitial pneumonia in the lung of a juvenile male. Pulmonary septa are expanded by lymphocytes, plasma cells, macrophages, and rare neutrophils. 20×, HE. **F** Subpleural focus of predominantly homogeneous alveolar aggregates of foamy macrophages (endogenous lipid pneumonia) in the lung of an adult male. 20×, HE.

The liver demonstrated the highest lesion burden, with 194 histopathological alterations documented across 56 ERBs (81.2%; 56/69). Glycogen accumulation progressing to vacuolar hepatopathy of glycogen type was observed in 81.2% (56/69) of specimens and has been previously characterized as a common incidental finding in captive ERB populations (Figure 2A) [16, 123, 124]. Given the brief interval between capture and euthanasia, it is unlikely that dietary alterations contributed to this finding [125, 126]. Mild-to-moderate, multifocal, lymphoplasmacytic hepatitis with occasional histiocytic components was identified in 74% of ERBs (51/69) (Figure 2B, C), frequently accompanied by predominantly mild necrosis (60.9%; 42/69) and hemorrhage (47.8%; 33/69). Among ERBs with hepatitis, 51% demonstrated RT-qPCR positivity for MARV in splenic tissue, while 49% (25/51) were negative. Evaluation of hepatic iron accumulation using Perls’ Prussian blue histochemical staining revealed hemosiderosis in 40.6% (28/69) of this free-ranging cohort, further classified as mild (21.7%; 15/69) or moderate (18.8%; 13/69) [98]. Additional hepatic findings included nodular hyperplasia (4.3%; 3/69), extramedullary hematopoiesis (2.9%; 2/69), sinusoidal inflammatory cell infiltrates (2.9%; 2/69), biliary ectasia (1.4%; 1/69), biliary hyperplasia (1.4%; 1/69), centrilobular degeneration (1.4%; 1/69), bridging fibrosis (1.4%; 1/69), and telangiectasia (1.4%; 1/69).

### Multiorgan perivascular cellular aggregates

**Figure 3:**
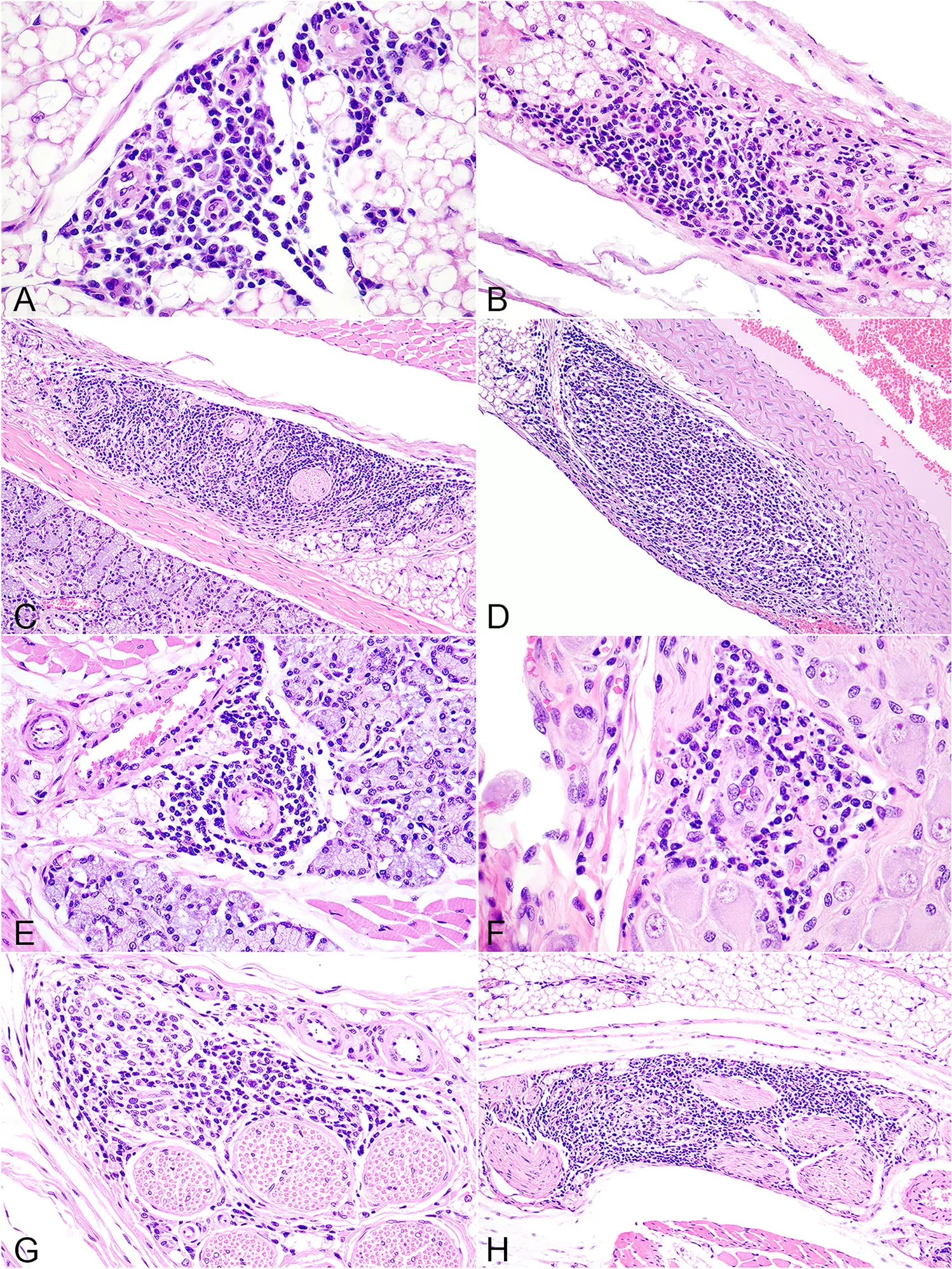
**Systemic perivascular inflammatory aggregates in Egyptian rousettes** (***Rousettus aegyptiacus***). Perivascular aggregates of predominantly lymphocytes, plasma cells, and fewer macrophages in **A** lingual adipose, juvenile male, 60×, HE. **B** pelvic subcutaneous adipose, adult female, 40×, HE. **C** adipose between orbicularis oculi muscle and parotid salivary gland, juvenile female, 20×, HE. **D** adipose adjacent to aorta at heart base, juvenile male, 20×, HE. **E** adipose within perianal gland, adult female, 40×, HE. **F** ganglia adjacent to the submandibular salivary gland, juvenile female, 60×, HE. **G** peripheral nerve in buccal subcutaneous adipose, juvenile male, 40×, HE. **H** peripheral nerve in caudal pelvic subcutaneous adipose, adult female, 20×, HE.

The most prevalent histopathological alteration consisted of variably sized perivascular aggregates composed predominantly of lymphocytes, plasma cells, and occasional histiocytes, with 232 discrete lesions affecting one (15.9%; 11/69) or multiple (65.2%; 45/69) organs in 56 ERBs (81.2%; 56/69). These cellular aggregates demonstrated highest prevalence in adipose tissue (154/232 lesions [66.4%], affecting 47/69 ERBs [68.1%]) (Figure 3A–E), followed by neural tissues (41/232 lesions [17.7%], affecting 26/69 ERBs [37.7%]) (Figure 3F, G, H). Detailed organ distribution and severity classifications are provided in Table 1.

**Table 1:** **Organ locations and severity of perivascular cellular aggregates in free-ranging Egyptian rousettes (*Rousettus aegyptiacus*) from Uganda**

Organ (no. of bats)	Severity (mild/moderate/severe)	Total number
Adipose (48)	86/48/20	154
Nerves (26)	21/14/6	41
Tongue (10)	6/4/0	10
Heart (8)	6/2/0	8
Salivary tissue (3)	3/0/0	3
Kidney (2)	2/0/0	2
Liver (2)	2/0/0	2
Lung (2)	2/0/0	2
Wing (2)	2/0/0	2
Adrenal gland (1)	0/1/0	1
External ear; ceruminous gland (1)	0/0/1	1
Great vessels (1)	1/0/0	1
Mammary gland (1)	1/0/0	1
Pancreas (1)	0/1/0	1
Teat (1)	1/0/0	1
Trachea (1)	0/1/0	1
Urinary bladder (1)	1/0/0	1

Variably sized perivascular aggregates composed predominantly of lymphocytes, plasma cells, and occasional histiocytes were present in one (15.9%; 11/69) or more (65.2%; 45/69) organs in 56 (81.2%, 56/69) ERBs.

### Dermatological pathology

A total of 39 ERBs (56.5%; 39/69) exhibited 67 cutaneous lesions characterized by perivascular to interstitial dermatitis of mild-to-severe intensity. Lesions were variably characterized by lymphoplasmacytic infiltrates (59.7%; 40/67 lesions) (Figure 2D), epidermal erosion with serocellular crusting (17.9%; 12/67), neutrophilic infiltrates (7.5%; 5/67), granulomatous or abscess formation (4.5%; 3/67), pyogranulomatous inflammation (4.5%; 3/67), epidermal hyperplasia/acanthosis (3.0%; 2/67), or epidermal ulceration (3.0%; 2/67). The majority of lesions were identified in whole-body, cephalic, or pelvic cross-sections; however, this distribution may reflect sampling methodology rather than authentic anatomical predilection.

### Pulmonary pathology

Respiratory system alterations (35 lesions in 26 ERBs [37.7%; 26/69]) were predominantly characterized by mild pulmonary hemorrhages (45.7%; 16/35 lesions), likely attributable to heightened cardiopulmonary stress during capture or iatrogenic trauma during intracardiac exsanguination. Mild, chronic interstitial pneumonia represented the most common inflammatory lesion (22.9%; 8/35) (Figure 2E), accompanied by mixed inflammatory pneumonia (8.6%; 3/35), embolic pneumonia (5.7%; 2/35), and focal endogenous lipid pneumonia (2.9%; 1/35) (Figure 2F). Additional pulmonary findings included mild interstitial fibrosis (5.7%; 2/35) and mild-to-moderate, subacute to chronic pleuritis (5.7%; 2/35).

### Salivary gland pathology

**Figure 4:**
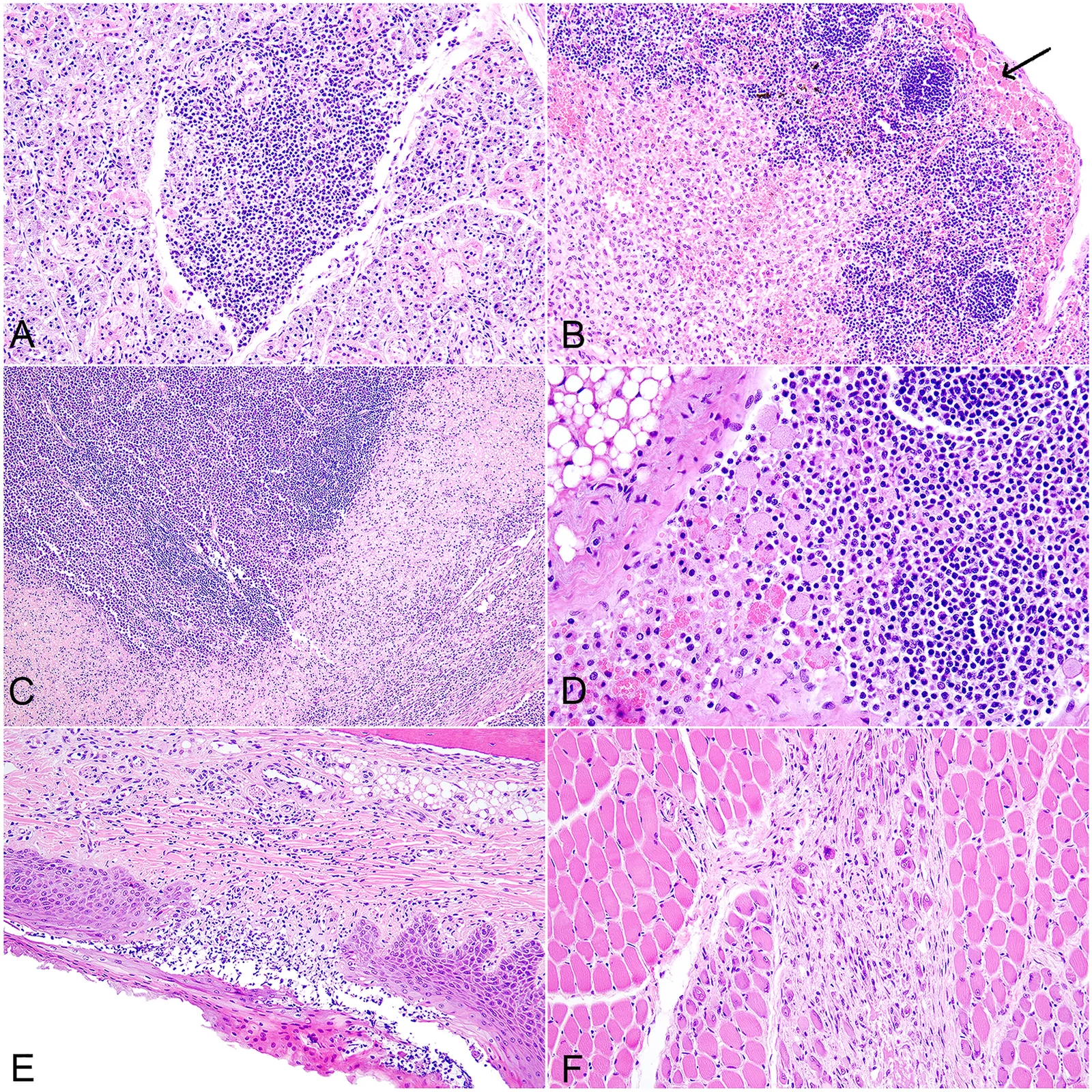
**Histopathologic findings in the salivary gland, lymph nodes, gingiva, and skeletal muscle in Egyptian rousettes** (***Rousettus aegyptiacus***). **A** Lymphoplasmacytic sialadenitis of the parotid salivary gland in a juvenile female. 20×, HE. **B** Erythrocyte-laden macrophages expand the subcapsular sinus (thin black arrow), and numerous erythrocytes expand the cortex and paracortex in a juvenile male. 20×, HE. **C** Severe neutrophilic and histiocytic lymphadenitis and cellulitis with necrosis, obliterating lymph node and adjacent connective tissue parenchyma in a juvenile female. 10×, HE. **D** Subcapsular foamy histiocytosis in a lymph node of an adult female. 40×, HE. **E** Focal ulceration and mucosal loss of the hard palate gingiva, with neutrophilic and histiocytic gingivitis and parakeratotic hyperkeratosis in a juvenile female. 20×, HE. **F** Skeletal myocyte degeneration with regeneration, replacement fibrosis, and infiltrating neutrophils and macrophages in a juvenile female. 20×, HE.

A total of 24 specimens with salivary gland alterations (34.8%; 24/69) demonstrated mild-to-moderate, multifocal, lymphoplasmacytic sialadenitis (100%; 24/24) (Figure 4A). Additional findings included mild, multifocal, periductal fibrosis in two specimens (8.3%; 2/24) and macrophage infiltration admixed within lymphoplasmacytic populations in one specimen (4.2%; 1/24).

### Lymphoid pathology

For analytical purposes, lymph nodes were categorized as “axillary” or “other” anatomical locations. Among 21 specimens (30.4%; 21/69) with axillary lymph node alterations, 11 demonstrated mild-to-moderate sinus erythrocytosis (40.7%; 11/27 total axillary lymph node lesions) (Figure 4B). Additional changes encompassed mild, multifocal mineralization of follicular germinal centers (37.0%; 10/27), erythrophagocytosis (7.4%; 2/27), follicular hyperplasia (7.4%; 2/27), and moderate-to-severe lymphadenitis, characterized either by prominent subcapsular histiocytosis (3.7%; 1/27) or subacute, necrotizing, neutrophilic adenitis with granuloma formation, severe pyogranulomatous and mononuclear cellulitis, and reactive endothelium (3.7%; 1/27) (Figure 4C). Alterations in all other examined lymph nodes (15.9%; 11/69) included moderate-to-severe, necrotizing, neutrophilic and/or mononuclear cellulitis and lymphadenitis (36.8%; 7/19 total lesions), mild-to-moderate phagocytosis (21.0%; 4/19), mild sinus erythrocytosis (15.8%; 3/19), mild-to-moderate fibrosis (10.5%; 2/19), germinal center mineralization (5.3%; 1/19), subcapsular foamy histiocytosis (5.3%; 1/19) (Figure 4D), and edema (5.3%; 1/19).

### Renal pathology

A total of 16 ERBs (23.2%; 16/69) exhibited renal alterations, with mild-to-moderate, lymphoplasmacytic, interstitial nephritis representing the most prevalent lesion (48.1%; 13/27 total lesions). Additional findings included mild tubular ectasia with proteinosis or granular casts (18.5%; 5/27), intracellular pigment deposition most commonly within proximal tubular epithelium (11.1%; 3/27), mild focally extensive chronic infarction (11.1%; 3/27), mild interstitial fibrosis (11.1%; 3/27), and mild tubular mineralization (3.7%; 1/27).

### Wing membrane pathology

A total of 14 ERBs (20.3%; 14/69) demonstrated predominantly inflammatory alterations within wing membrane structures, most commonly affecting the plagiopatagium and/or dactylopatagium. Neutrophilic and eosinophilic dermatitis, fibrosis, hyperkeratosis, and acanthosis (each 85.7%; 12/14 total lesions) represented the most frequent findings and were commonly associated with arthropod parasite presence, either within presumptive hair follicles or within the stratum corneum. Additional wing membrane findings included mild perivascular to interstitial lymphoplasmacytic dermatitis (28.6%; 4/14), epidermal ulceration (28.6%; 4/14), and mild histiocytic dermatitis (7.1%; 1/14).

### Gingival pathology

A total of 11 ERBs (15.9%; 11/69) exhibited gingival alterations, most commonly lymphoplasmacytic gingivitis (45.5%; 5/11 total lesions), neutrophilic gingivitis (9.1%; 1/11), or ulcerative gingivitis (9.1%; 1/11) (Figure 4E), occasionally accompanied by intraepithelial pustule formation (27.3%; 3/11).

### Skeletal muscle pathology

A total of 11 ERBs (15.9%; 11/69) demonstrated 19 skeletal muscle alterations, including mild-to-moderate, focally extensive to multifocal fibrosis (31.6%; 6/19), degenerative myopathies (26.3%; 5/19), regenerative myopathies (21%; 4/19) (Figure 4F), and mild-to-moderate myositis ranging from lymphoplasmacytic (10.5%; 2/19) to eosinophilic (5.3%; 1/19) or histiocytic (5.3%; 1/19) infiltration patterns.

### Adrenal gland pathology

**Figure 5:**
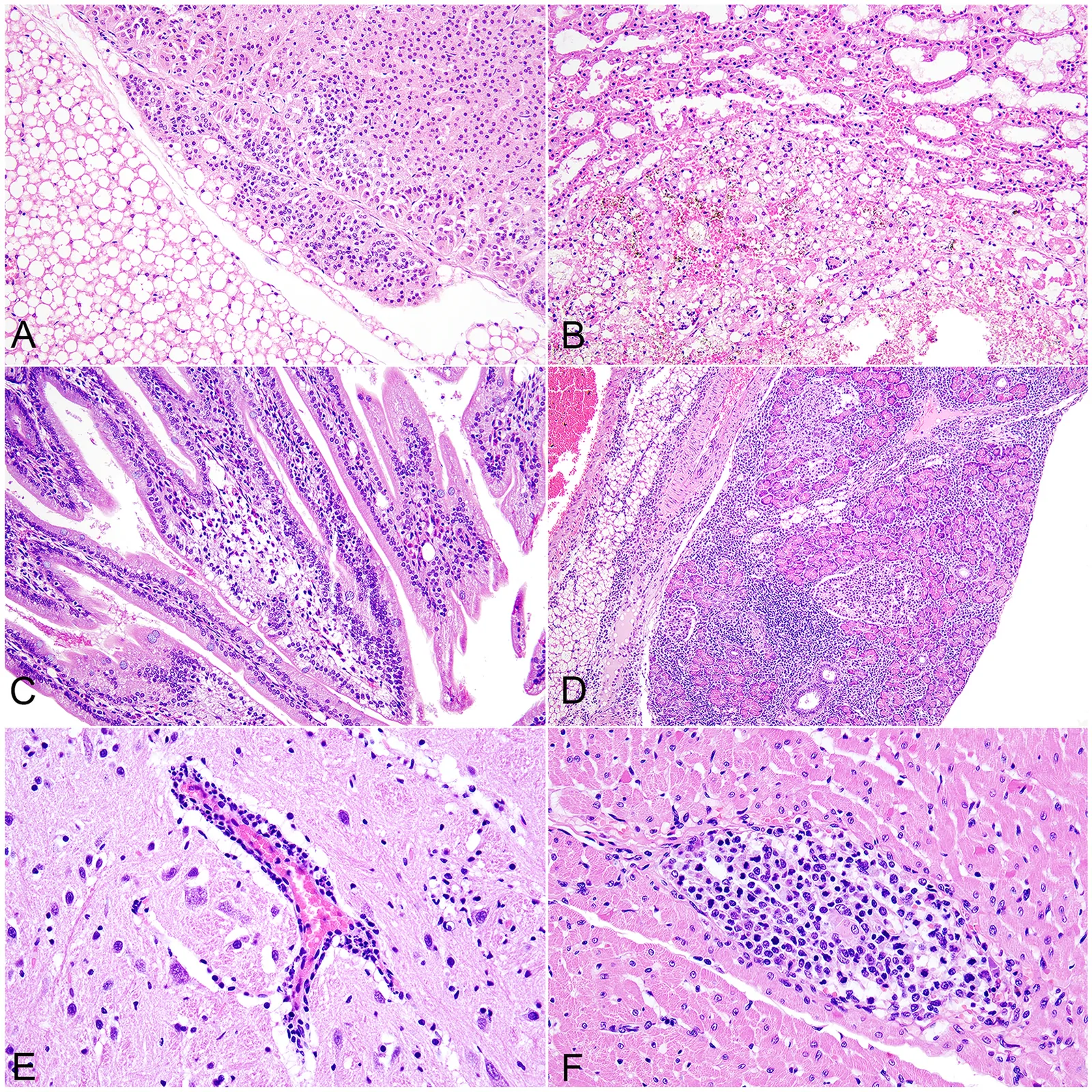
**Pathologic histologic findings in the adrenal gland, mammary gland, small intestine, pancreas, brain, and heart in Egyptian rousettes** (***Rousettus aegyptiacus***). **A** Adrenal cortical hyperplasia in an adult male. 20×, HE. **B** Necrotizing mastitis with neutrophils, macrophages, multinucleated giant cells, and hemorrhage in an adult female. 20×, HE. **C** Lymphoplasmacytic enteritis with villous edema and occasional neutrophils in a juvenile female. 20×, HE. **D** Lymphoplasmacytic, histiocytic, and neutrophilic pancreatitis with necrosis in a juvenile male. 10×, HE. **E** Lymphoplasmacytic perivascular cuffing in the brainstem of a juvenile female. 40×, HE. **F** Lymphoplasmacytic and histiocytic cardiomyositis with scant hemorrhage in the left ventricle of a juvenile male. 40×, HE.

Among nine ERBs with adrenal gland alterations, four demonstrated mild cortical hyperplasia (44.4%; 4/9 total lesions) (Figure 5A), while two exhibited mild extramedullary hematopoiesis within the adrenal medulla and/or cortex (22.2%; 2/9). Brown, finely granular intracellular pigment was observed within distal zona reticularis cells or proximal zona glomerulosa cells in three ERBs (33.3%; 3/9).

### Additional organ systems

Additional pathological changes affecting fewer than ten ERBs per organ system are comprehensively detailed in an additional file (see Additional file 2), encompassing mammary gland (11.6%; 8/69) (Figure 5B), nasal turbinates (10.1%; 7/69), external ear (8.7%; 6/69), small intestine (8.7%; 6/69) (Figure 5C), colon (5.8%; 4/69), pancreas (5.8%; 4/69) (Figure 5D), pharynx (2.9%; 2/69), prostate (2.9%; 2/69), adipose tissue (1.5%; 1/69), brain (1.5%; 1/69) (Figure 5E), cervix (1.5%; 1/69), eye (1.5%; 1/69), heart (1.5%; 1/69) (Figure 5F), thymus (1.5%; 1/69), tongue (1.5%; 1/69), trachea (1.5%; 1/69), and uterus (1.5%; 1/69).

### Parasitological findings

A total of 72 parasitic organisms were identified across 39 ERBs (56.5%; 39/69), with highest prevalence in haired skin Sects. (30.8%; 20/65) and deep external ear canal (26.2%; 17/65), followed by wing membrane (13.8%; 9/65), small intestine (12.3%; 8/65), stomach (7.7%; 5/65), nasal turbinates (6.2%; 4/65), gingiva (4.6%; 3/65), and singular occurrences in colon, esophagus, pancreas, extra-gastric tissues, adipose tissue, and urinary bladder (each 1.5%; 1/65). In numerous instances, parasitic degeneration precluded confident taxonomic identification even to family level, and histological descriptions of ERB-associated parasites are currently absent from the literature for comparative purposes. Blood smears were not prepared at the time of capture; therefore, evaluation for hemoparasites could not be performed as part of this study.

**Figure 6:**
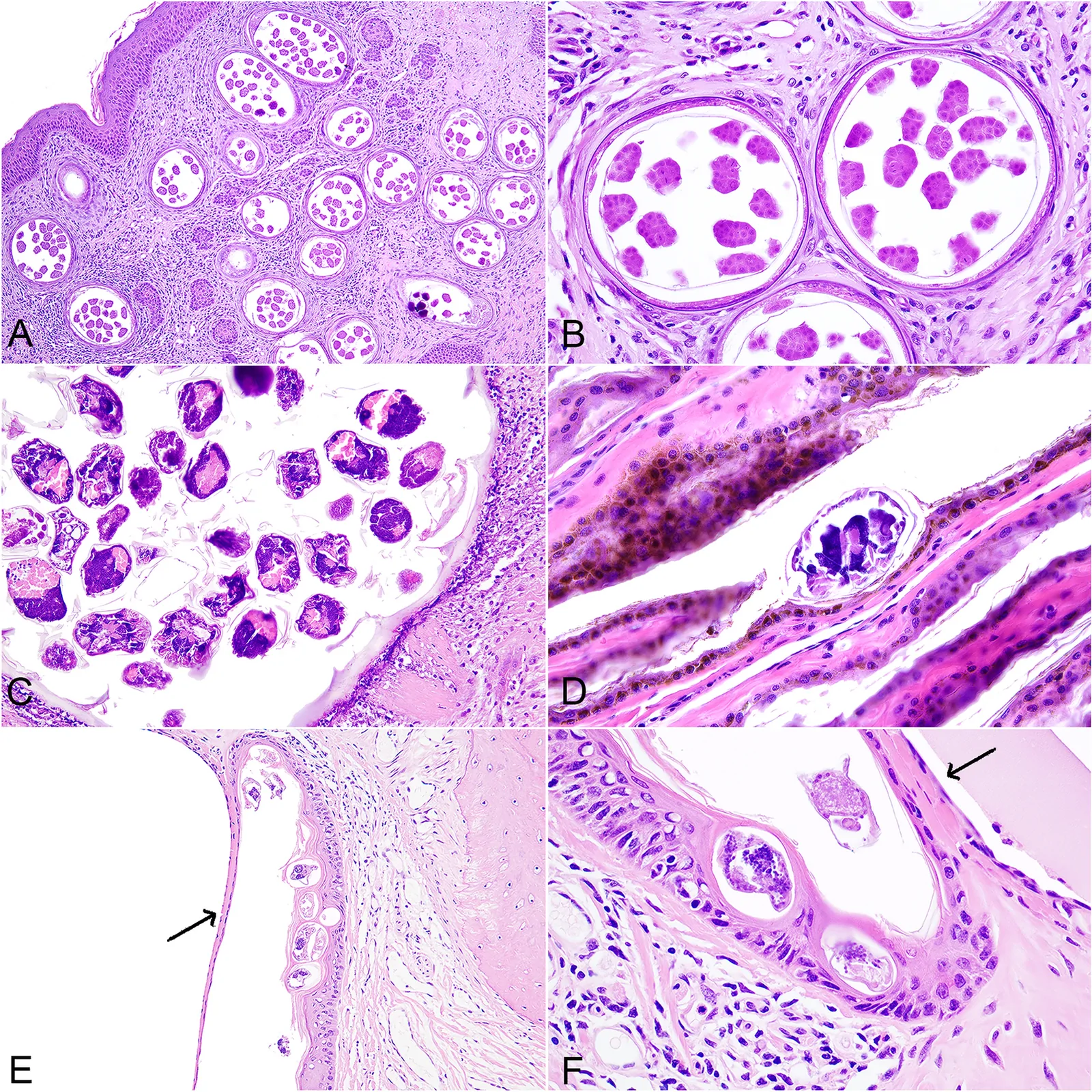
**Ectoparasites in Egyptian rousettes** (***Rousettus aegyptiacus***) **from Uganda**. **A**, **B** Numerous multifocal, 150–300 µm parasitic cysts containing ~20 µm arthropod eggs expanding the subepithelial connective tissue of the wing membrane of a juvenile male. **A** 10×, HE **B** 40×, HE. **C** 700 µm diameter parasitic cyst containing multiple, 60–90 µm arthropods (mites) expanding the subepithelial connective tissue of the wing membrane of a juvenile male. 20×, HE. **D** Ovoid, 60 × 100 µm, arthropod (mite) within the stratum corneum of the wing membrane epithelium of a juvenile male. 40×, HE. **E**, **F** Multiple, 25–45 µm arthropods (mites) with thin, ridged cuticles and striated musculature expanding the stratum corneum of the deep external ear canal. Mites are adjacent to the tympanic membrane (thin black arrow), within the deep external ear canal lumen of an adult female. **E** 20×, HE. **F** 60×, HE.

**Figure 7:**
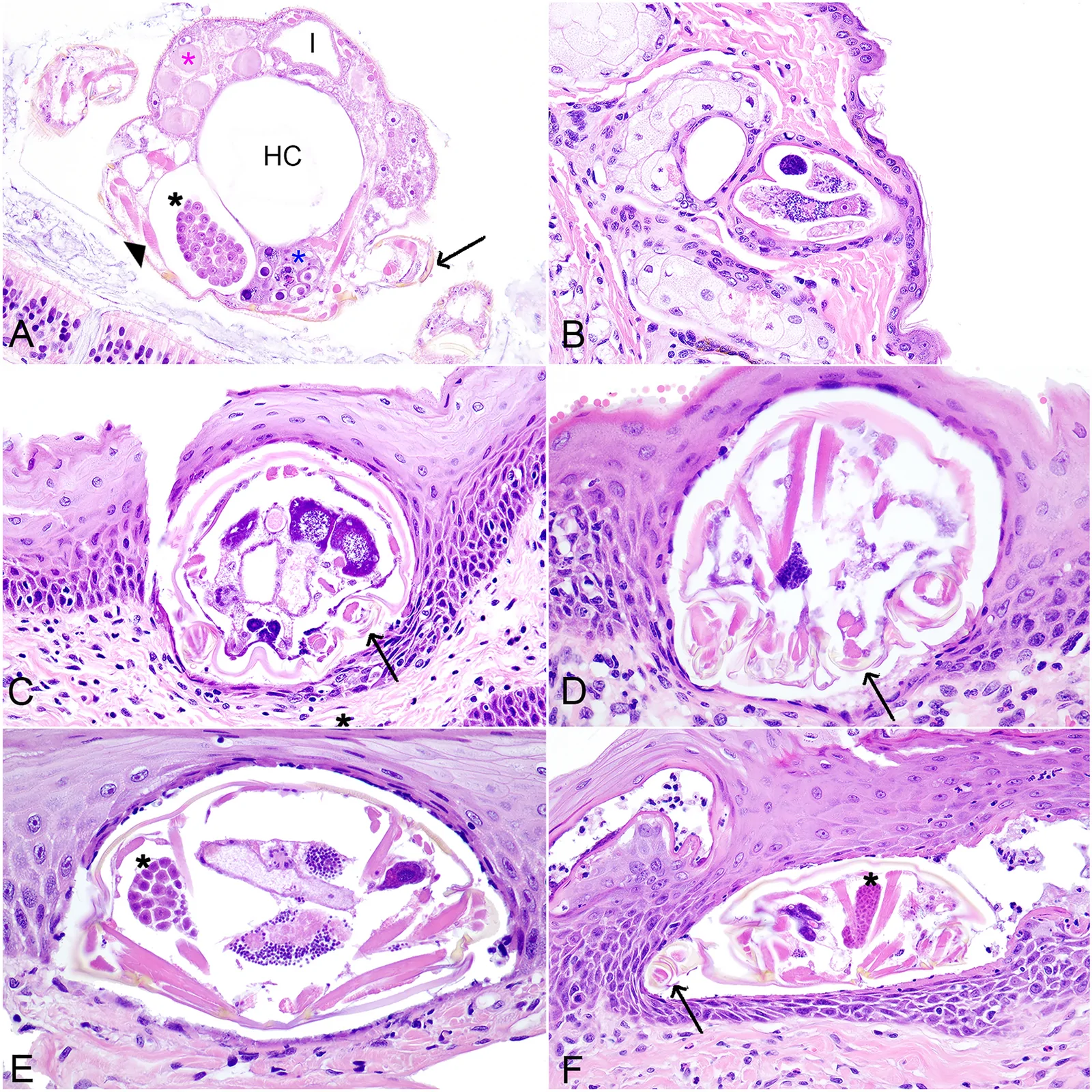
**Ectoparasites in Egyptian rousettes** (***Rousettus aegyptiacus***) **from Uganda**. **A** 180 × 250 µm arthropod with chitinized appendages and cuticular spines in the nasal turbinate lumen of a juvenile female. Note the chitinous exoskeleton, dorsal spines, jointed appendages (thin black arrow), striated muscle (black arrowhead), body cavity (hemocoel, HC), and intestinal and reproductive structures. Single intestinal tube lined by flattened epithelium with an apical brush border (I), sperm (black asterisk), ovaries (pink asterisk) and developing eggs (blue asterisk). 40×, HE. **B** 60 × 100 µm arthropod expanding the subepithelial adnexa of a juvenile male. 40×, HE. **C**–**F** Arthropods with jointed appendages, striated muscle, and cuticular spines expanding the stratum corneum of the **C** cranial dermis of a juvenile female; 150 µm diameter; 40×, HE. **D** perirectal dermis of a juvenile female; 130 µm diameter; 60×, HE. **E** cranial dermis of a juvenile female; 105 × 185 µm; 40×, HE. **F** buccal mucosa of a juvenile female; 75 × 190 µm; 20×, HE.

**Figure 8:**
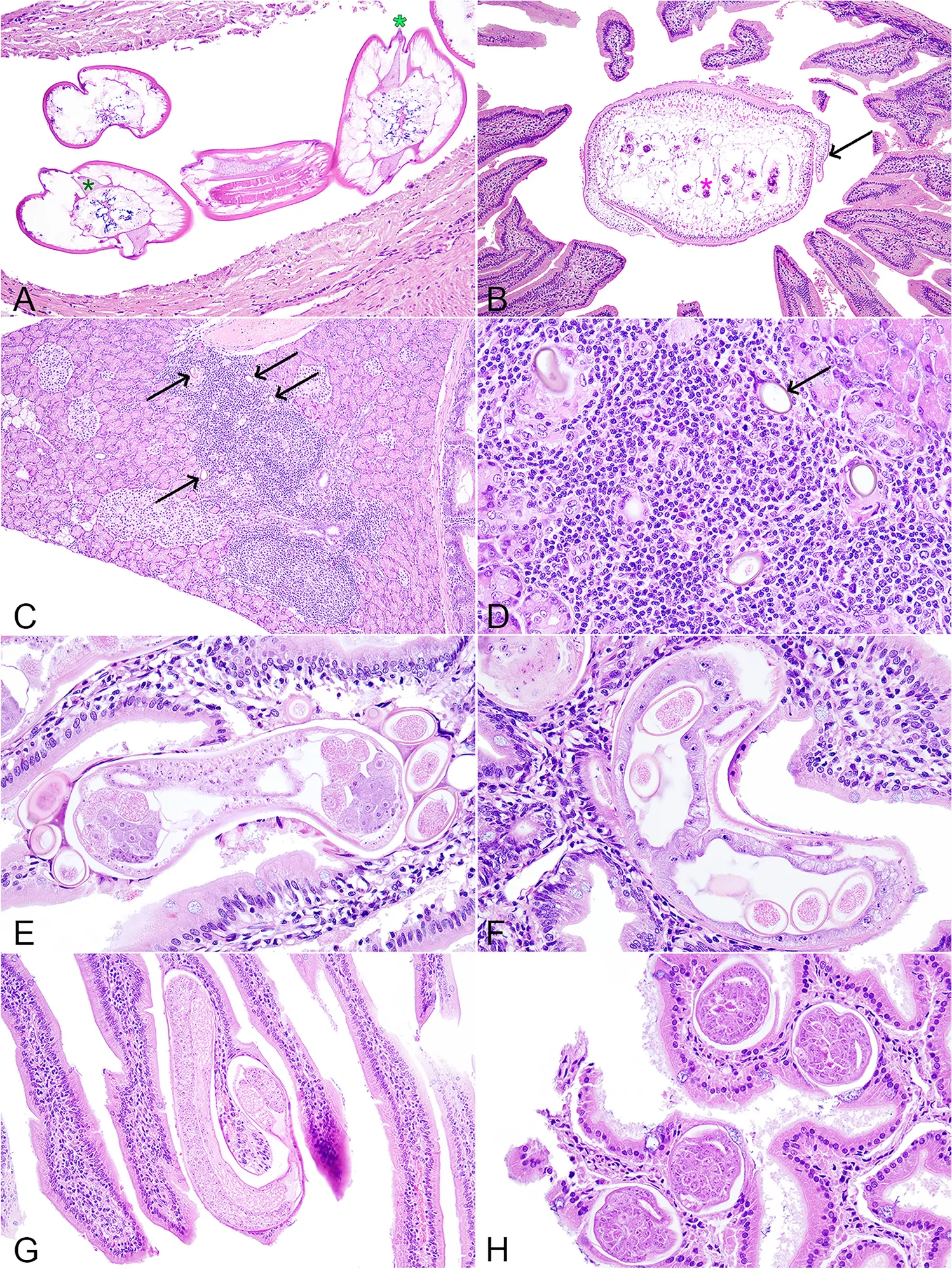
**Endoparasites in Egyptian rousettes** (***Rousettus aegyptiacus***) **from Uganda**. **A** 175 × 200 µm larval ascarids with lateral alae (lime green asterisk), prominent lateral chords (forest green asterisk), and coelomyarian musculature within a 0.5 × 1.2 mm extragastric granuloma of an adult male; 20×, HE. **B** Craspedote cestode with lateral vellum (thin black arrow) and central uterus with degenerate eggs (pink asterisk) in the small intestinal lumen of a juvenile male; 10×, HE. **C**, **D** Focally extensive section of lymphoplasmacytic and histiocytic pancreatitis with multiple trematode eggs (thin black arrows) with thick, yellow–brown shells in a juvenile male. **C** 4×, HE. **D** 20×, HE. **E**, **F** Aphasmid nematode with multiple eggs with rare bipolar plugs expanding small intestinal villi in a juvenile female; 40×, HE. **G** Aphasmid nematode expanding a small intestinal villus of a juvenile male; 20×, HE. **H** Cross sections of aphasmid nematodes expanding small intestinal villi of a juvenile female; 40×, HE.

Numerous variably sized organisms were identified within subepithelial connective tissue or stratum corneum of wing membranes, most frequently within the plagiopatagium (Figure 6A–D), as well as deep within external ear canals, often positioned immediately adjacent to tympanic membranes (Figure 6E, F). These organisms were characterized by thin, ridged cuticles and striated musculature, consistent with mite species, possibly *Psorergatoides* spp. [127–130]. Organisms within haired skin sections were frequently located within the stratum corneum and characterized by jointed appendages and cuticular spines (Figure 7B–E), representing diagnostic features of Sarcoptidae mites, a family previously documented in ERB populations [85, 131, 132]. Additional parasitic organisms were identified across various tissues, including an arthropod with chitinized appendages and cuticular spines in the nasal turbinate lumen (Figure 7A), larval ascarids with lateral alae and prominent lateral chords within extra-gastric granulomas (Figure 8A), craspedote cestodes with lateral vellum within small intestinal lumens (Figure 8B), trematode eggs within pancreatic tissue (Figure 8C, D), and aphasmid nematodes expanding small intestinal villi (Figure 8E, H).

## Discussion

This investigation represents the first comprehensive characterization of spontaneous pathological processes in free-ranging Egyptian rousettes from Uganda, providing unprecedented insights into the spectrum of naturally occurring disease manifestations within this epidemiologically critical reservoir species. The study cohort was originally procured during efforts to identify filoviral reservoir hosts, thereby affording a unique opportunity to investigate spontaneous disease processes within an ostensibly healthy population. Through systematic evaluation of 69 specimens, 794 discrete histological lesions were documented across 28 organ systems, with gross pathological abnormalities identified in more than 85% of examined individuals. Overall, there were no significant differences between MARV-positive and MARV-negative bats in this study.

### Hepatic pathology and viral associations

Hepatic glycogen-type vacuolar hepatopathy and multifocal lymphoplasmacytic hepatitis likely represent physiological adaptations and MARV-associated or other infection-related hepatopathological manifestations, respectively. These findings were anticipated given the well-documented propensity of ERBs for physiological hepatic glycogen accumulation and the fact that 51% of specimens demonstrated RT-qPCR positivity for MARV in splenic tissue at capture. The 1622 ERBs captured in Uganda during 2008–2009 constituted components of a comprehensive MARV ecological investigation [9]; the MARV-positive specimens included in this histological analysis represent a targeted subset of that cohort, specifically retained due to their viral positivity status. This subset therefore does not represent natural infection prevalences in free-ranging juvenile ERB populations, which approximate 2.5% [9].

The mild-to-moderate, multifocal lymphoplasmacytic hepatitis with occasional histiocytic inflammation, mild necrosis, and hemorrhage observed in this cohort closely mirrors the histopathologic lesions described in experimentally MARV-infected ERBs. These lesions are characterized by mild, transient hepatitis with small, randomly scattered aggregates of macrophages and lymphocytes, occasional neutrophils, necrotic or apoptotic hepatocytes, karyorrhectic debris, and minimal hemorrhage [16]. The concordance between natural and experimental lesions suggests that naturally occurring infections elicit comparable hepatic immune responses, reinforcing the biological relevance of experimental ERB models for understanding reservoir host–virus interactions and pathogenesis. Notably, 49% (25/51) of free-ranging ERBs were MARV PCR-negative yet showed hepatopathology, potentially reflecting false-negative PCR results or infection with another hepatotropic virus, such as Kasokero virus [123].

### Systemic perivascular inflammatory aggregates

The most prevalent histological finding was the presence of perivascular inflammatory aggregates, documented across 17 distinct organ systems, with predominant manifestation in adipose tissue and neural-adjacent locations. No overarching pattern in their anatomical distribution was discernible, and their apparent overrepresentation in specific anatomical sites, including the cardiac base, mesentery, and pelvic regions, may reflect sampling methodology bias. This finding has not been documented in ERBs evaluated from research colony or zoological managed care environments [97, 98]. Perivascular inflammation represents a common, nonspecific histological manifestation associated with diverse infectious and noninfectious processes across a wide range of vertebrate taxa. These encompass viral diseases (e.g., rabies virus [133], canine distemper virus [134], feline infectious peritonitis [135], West Nile virus [136]); bacterial infections (e.g., listeriosis [137], rickettsiosis [138]); parasitic infections (e.g., toxoplasmosis [139], equine protozoal myeloencephalitis [140]); fungal infections (e.g., cryptococcosis [141]); immune-mediated diseases (e.g., granulomatous meningoencephalitis [142]); environmental toxicities (e.g., lead toxicosis [143], hairy vetch toxicosis [144]); and vascular or autoimmune disorders (e.g., atherosclerosis [145], pulmonary hypertension [145], systemic lupus erythematosus, and rheumatoid arthritis [146]). The mechanistic basis underlying aggregate formation remains unclear and may reflect chronic antigenic stimulation, subclinical exposures to viral or parasitic agents, systemic immune surveillance mechanisms, or environmental stressor responses. Future investigations examining potential roles of infectious pathogens, metabolic or oxidative stress, or ectoparasitic burden in systemic perivascular aggregate development are warranted.

### Additional pathological manifestations

Additional lesions, including dermatitis, interstitial pneumonia, sialadenitis, and mild nephritis, further underscore the complexity of host–pathogen–environment interactions within free-ranging chiropteran populations. While many of these lesions are unlikely to constitute clinically significant entities in isolation, they may exert cumulative effects on host health status, particularly in juvenile or immunocompromised individuals.

### Parasitological discoveries and implications

This investigation provides the first macroscopic and microscopic documentation of wing membrane and deep external ear canal mites, morphologically consistent with *Psorergatoides* sp., which have been previously reported in *Myotis macropus* populations in Australia [129]. Additional ectoparasitic and endoparasitic organisms were documented affecting numerous anatomical systems. Enhanced characterization of ERB endoparasitic and ectoparasitic fauna is warranted, as parasitic infections may modulate host immunity and alter concomitant infection dynamics [147, 148]. Uncharacterized ectoparasites could assume important roles in endemic pathogen transmission or maintenance, as they represent well-established vectors for numerous diseases [149, 150]. This principle has been demonstrated in ERB populations, where *Ornithodoros* (*Reticulinasus*) *faini* ticks maintain Kasokero virus through enzootic transmission cycles [28, 79, 123, 151]. Future investigations should prioritize robust field necropsy methodologies to ensure appropriate parasite collection for macroscopic and microscopic evaluation, including comprehensive external ear canal dissection and intact specimen preservation in 70–90% ethanol [150].

### Absence of fungal and neoplastic pathology

Notably, no fungal pathogens were identified within this cohort. However, due to undocumented fixative transfers and carcass manipulations since initial collection circa 2008, grossly appreciable fungal elements may no longer have been evident and therefore not selected for histological evaluation. All previously documented fungal pathogens in ERBs have been identified in managed care populations (see Additional file 1).

Additionally, no neoplastic processes were identified within this cohort, consistent with low neoplasia rates in managed care ERB populations. Generally, bats represent long-lived, tumorigenesis-resistant species, with recent research suggesting that evolutionary adaptations and genomic characteristics supporting this capacity include negative selection against non-long terminal repeat (non-LTR) retrotransposon genomic accumulation [152], differential expression of telomerase maintenance genes [ 153], positive selection for multiple tumor suppressor and DNA-repair genes [154], and potentially efficient prevention of oxidative stress-induced damage [155].

### Study limitations and methodological considerations

This investigation is not without limitations. Samples were analyzed over a decade post-collection, and preservation artifacts may have impacted parasitic morphology. The study was constrained to histological techniques without parallel molecular diagnostic incorporation, which would have enhanced etiological investigation capabilities. Molecular investigations to identify potential pathogens within affected tissues were not feasible due to nucleic acid degradation and intractable contaminants that could not be eliminated during nucleic acid extraction and sample cleanup procedures. Consequently, the extent to which viral pathogens contributed to identified inflammatory lesions remains undetermined. Immunohistochemical staining was not attempted due to contaminants identified during extraction efforts and prolonged fixative exposure. Application of special stains (Gram stain, Giemsa stain) did not elucidate bacterial pathogen presence in lesions characterized by marked neutrophilic and/or histiocytic infiltrates. Additionally, the population sample demonstrated juvenile skewing, and temporal or seasonal effects on disease prevalence could not be assessed. A further limitation was splenic tissue unavailability for histological evaluation, as it was prioritized for Marburg virus RT-qPCR at capture. Future investigations should prioritize fresh tissue sample retention for unbiased pathogen discovery using platforms such as Virocap [156] while reserving splenic tissue for diagnostic and histopathological assessment.

## Future research directions

Beyond previously mentioned topics, this investigation opens several promising research avenues. First, systematic molecular characterization of lesions, particularly those involving lymphoid organs, hepatic tissue, and integumentary systems, could facilitate underlying pathogen identification and disease etiology clarification. Second, comprehensive investigations into immunological consequences of systemic and chronic low-level inflammation, such as the perivascular infiltrates observed herein, may illuminate how ERBs balance viral disease tolerance with immune control mechanisms. Third, enhanced understanding of parasitic diversity, life cycles, and vector competence within ERB populations is needed, especially considering ectoparasitic roles in pathogen transmission. Finally, longitudinal studies incorporating health status, reproductive condition, and seasonal variation could help delineate interactions between disease burden, host fitness, and spillover potential.

## Conclusions and broader implications

This investigation provides the first tissue-based characterization of naturally occurring pathological processes in free-ranging ERBs, a critical natural reservoir host for orthomarburgviruses [9, 15, 16, 21], demonstrating that even outwardly healthy individuals harbor extensive histological lesions, many with probable infectious or inflammatory origins. These findings underscore the importance of characterizing reservoir host health beyond simple pathogen presence or absence and highlight the necessity for integrated approaches combining pathology, molecular biology, and ecology to enhance understanding of zoonotic disease spillover dynamics and inform strategies for mitigating future outbreaks in human and other susceptible host populations.

## Supplementary Information


**Additional file 1. Pathogens and parasites reported in Egyptian rousette bats** ***(Rousettus aegyptiacus)***.S = Serology, M = Molecular biology, ^#^ = Antibodies in serum, ^*^ = found in feces under roosting *ERB* colonies, ^@^ = described in *Rousettus aegyptiacus occidentalis*, ^$^ = possibly accidental, ^&^ = described in *Rousettus aegyptiacus leachi*

## Data Availability

No datasets were generated or analyzed during the current study.
