# Supplementary material for: Characterization of spontaneous gross and histologic findings in free-ranging Egyptian rousettes (Rousettus aegyptiacus) from Uganda with a review of reported pathogens
Source: Vet Res. 2026 Aug 27;57:155. doi: 10.1186/s13567-026-01785-z (PMC13523373; doi:10.1186/s13567-026-01785-z)
Supplement: Supplementary file 1 [file 13567_2026_1785_MOESM1_ESM.docx]

**Additional File 1: Pathogens and parasites reported in Egyptian rousette bats (*Rousettus aegyptiacus).*** S = Serology, M = Molecular biology, ^#^ = Antibodies in serum, * = found in feces under roosting *ERB* colonies, ^@^ = described in *Rousettus aegyptiacus occidentalis,* ^$^ = possibly accidental*,* ^&^ = described in *Rousettus aegyptiacus leachi*

| **Group** | **Family** | **Pathogen** | **Detection Method** | **Location** | **Ref** |
| --- | --- | --- | --- | --- | --- |
| **Virus (RNA)** | *Astroviridae* | Astrovirus sp. | M | Gabon, Kenya | [1, 2] |
|  | *Caliciviridae* | Calicivirus sp. | M | Kenya | [2] |
|  | *Coronaviridae* | Betacoronavirus sp. | M | Kenya | [3] |
|  |  | Coronavirus sp. | M | Guinea, Kenya, South Africa | [2, 4-7] |
|  | *Filoviridae* | Zaire ebolavirus | S | Gabon | [8] |
|  |  | Orthomarburgvirus marburgense (Ravn virus) | S, M | South Africa, Uganda | [9-11] |
|  |  | Orthomarburgvirus marburgense (Marburg virus) | S | Gabon | [8-17] |
|  | *Flaviviridae* | Uganda S virus | S | Uganda | [18] |
|  |  | Entebbe bat virus | S | Kenya | [19] |
|  |  | Israel turkey meningoencephalitis virus | S | Israel | [20] |
|  |  | Ntaya virus | S | Kenya | [19] |
|  |  | Usutu virus | S | Kenya | [19] |
|  |  | Pegivirus | M | Kenya, Nigeria | [21] |
|  |  | West Nile virus | S | Kenya, Israel | [19, 20] |
|  |  | Yellow fever virus | S | Uganda | [22] |
|  |  | Zika virus | S | Uganda | [22, 23] |
|  | *Nairoviridae* | Kasokero virus | S | Uganda | [24] |
|  |  | Yogue virus | S, M | Senegal | [25] |
|  |  | Crimean-Congo hemorrhagic fever orthonairovirus | S | Gabon | [26] |
|  | *Orthomyxoviridae* | Influenza A virus | M | Egypt | [27] |
|  | *Orthoreoviridae* | Orthoreovirus | M | Zambia | [28, 29] |
|  | *Paramyxoviridae* | Hendra(-like) henipavirus | S | Ghana | [30] |
|  |  | Nipah henipavirus | S | Ghana | [30] |
|  |  | Cedar henipavirus | S | Ghana | [30] |
|  |  | Sosuga pararubulavirus | S, M | Uganda, Sierra Leone | [31, 32] |
|  |  | Rubulavirus spp. | M | South Africa, Congo | [33, 34] |
|  |  | Sosuga pararubula(-like) virus | M | South Africa | [33] |
|  |  | Henipavirus sp. | M | Gabon | [34] |
|  | *Peribunyaviridae* | Bunyamwera orthobunyavirus | S | Uganda | [22] |
|  | *Phenuiviridae* | Rift Valley Fever phlebovirus | S | Uganda^#^ | [35] |
|  | *Rhabdoviridae* | Lagos bat virus | M, S | Kenya, Nigeria^#^, and Togo, Egypt, France via “Africa” | [36-39] |
|  |  | European bat lyssavirus 1 | M | Denmark and Netherlands (managed care) | [40-43] |
|  |  | Shimoni bat virus | S | Kenya, Nigeria^#^ | [37, 39] |
|  |  | Mokola virus | S | Nigeria^#^ | [37] |
|  |  | Ikoma lyssavirus | S | Nigeria^#^ | [37] |
|  | *Sedoreoviridae* | Rotavirus A | M | Kenya, Zambia | [2, 44] |
|  |  | Bukakata orbivirus | M | Uganda | [45] |
|  | *Togoviridae* | Chikungunya virus | M | Senegal | [43] |
|  |  | Semliki Forest virus | S | Uganda | [22] |
|  |  | Alphavirus sp. | S | Uganda | [35] |
|  |  | O’nyong’nyong virus | S | No information | [46] |
| **Virus (DNA)** | *Adenoviridae* | Bat mastadenovirus | M | South Africa | [47] |
|  | *Herpesviridae* | Unclassified *Herpesviridae* | M | South Africa, Hungary (managed care) | [48, 49] |
|  |  | Betaherpesvirus sp.  Gammaherpesvirus sp. | M | Southern Spain (managed care), Zambia | [50, 51] |
|  | *Papillomaviridae* | Rousettus aegyptiacus papillomavirus type 1 | M | MI, USA (managed care) | [52, 53] |
|  | *Polyomaviridae* | Betapolyomavirus sp. | M | Zambia | [54] |
|  |  | Unclassified *Polyomaviridae* | M | Kenya | [55] |
|  | *Poxviridae* | Unclassified *Poxviridae* | M | Israel | [56, 57] |
| **Bacteria** | *Bartonellaceae* | *Bartonella rousetti* | M, S | SW Nigeria, Zambia | [58, 59] |
|  |  | *Bartonella* spp. | M | Kenya, South Africa | [60-62] |
|  | *Bifidobacteriaceae* | *Bifidobacterium* spp. | M | Italy (managed care) | [63] |
|  | *Borreliaceae* | *Borrelia* sp. | M | Zambia | [64] |
|  | *Enterobacteriaceae* | *Escherichia* sp. | M | Republic of Congo | [65] |
|  |  | *Kluyvera ascorbata* | S | South Korea (managed care) | [66] |
|  | *Leptospiraceae* | *Leptospira interrogans* | M | South Africa | [67] |
|  |  | *Leptospira borgpetersenii* (or -like) | M | South Africa | [67] |
|  | *Mycoplasmataceae* | *Mycoplasma* sp. | M | Northern Nigeria | [68] |
|  | *Staphylococcaceae* | *Staphylococcus aureus* | M | Gabon, Israel | [69, 70] |
|  |  | *Staphylococcus schweitzeri* | M | Gabon | [69] |
|  | *Yersiniaceae* | *Yersinia pseudotuberculosis* | S, M | New York and Japan (managed care) | [71, 72] |
| **Fungal** | *Ajellomycetaceae* | *Histoplasma sp.* | M | United States (managed care) | [73, 74] |
|  | *Cryptococcaceae* | *Cryptococcus sp.* | Unk | Unknown (managed care) | [74] |
|  | *Pneumocystidaceae* | *Pneumocystosis sp.* | M | France (managed care) | [75] |
|  | *Unikaryonidae* | *Encephalitozoon hellem* | M | United States (managed care) | [76] |
| **Protozoa** | *Eimeriidae* | *Eimeria rousetti* | Microscopy | Egypt | [77] |
|  | *Gregarinidae* | *“Gregarina rousetti n. sp.”* | Microscopy | Egypt | [78] |
|  | *Plasmodiidae* | *Hepatocystis* sp. | M | North-Central Nigeria | [79] |
|  |  | *Plasmodium rousetti* | S | Ghana, Congo | [80, 81] |
|  |  | *Plasmodium voltaicum* | S | Guinea, Liberia, Ivory Coast | [82] |
|  | *Trypanosomatidae* | *Trypanosoma* spp. | M | Gabon, South Africa | [83, 84] |
| **Nematode** | Filariidae | *Diptalonema vitae* |  | Egypt | [85] |
| **Arthropod (“bed bugs”)** | Cimicidae | *Afrocimex leleupi* |  | Congo | [81] |
|  | Cimicidae | *Afrocimex constrictus* |  | Kenya | [86] |
| **Arthropod (Crustacean)** | Phylum Pentastomida |  |  | Uganda | [11] |
| **Arthropod (fleas)** | Pulicidae | *Archaeopsylla metallescens* |  | Egypt | [70] |
|  | Ischnopsyllidae | *Thaumapsylla breviceps* |  | South Africa | [70, 87] |
|  | Ischnopsyllidae | *Thaumapsylla breviceps orientalis* |  | Congo | [70, 81] |
| **Arthropod (flies)** | Hippoboscidae | *Basila nana* |  | Lebanon | [88] |
|  | Nycteribiidae | *Cyclopodia greefi greefi* |  | Ghana | [89] |
|  | Nycteribiidae | *Eucampsipoda aegyptia* |  | Egypt, UAE, Yemen, Oman, Iran, Jordan, Turkey, Lebanon, Palestine, Saudi Arabia | [70, 88, 90-92] |
|  | Nycteribiidae | *Eucampsipoda africana* |  | Gabon, South Africa, Republic of the Congo, Liberia, Zambia^&^ | [87, 91, 93-95] |
|  | Nycteribiidae | *Eucampsipoda africanum* |  | Congo, Ghana | [81, 89] |
|  | Nycteribiidae | *Eucampsipoda hyrtlii* |  | Iran, Turkey | [96, 97] |
|  | Nycteribiidae | *Eucampsipoda hyrtlii diversa* |  | Egypt, Israel, Lebanon | [70] |
|  | Nycteribiidae | *Nycteribia alternata* |  | Ghana | [89] |
|  | Nycteribiidae | *Nycteribia pedicularia* |  | Palestine, Israel, Lebanon | [70, 88, 90] |
|  | Nycteribiidae | *Nycteribia schmidlii* |  | Palestine | [90] |
|  | Nycteribiidae | *Nycteribia schmidlii scotti* |  | Gabon | [94] |
|  | Streblidae | *Brachytarsina* sp. |  | Egypt | [70] |
|  | Streblidae | *Brachytarsina (Nycteribosca) africana* |  | Congo | [81] |
|  | Streblidae | *Brachytarsina (Nycteribosca) allaudi* |  | Congo, Gabon | [81, 94] |
|  | Streblidae | *Brachytarsina bequaerti* |  | Tanzania | [98] |
|  | Streblidae | *Megastrebla (Aoroura) bequaerti* |  | Tanzania | [98] |
|  | Streblidae | *Nycteribosca bequaerti* |  | Tanzania | [98] |
| **Arthropod (mites)** | Acarinae | *Acarus* sp. |  | Ghana | [89] |
|  | Carpoglyphidae | *Carpoglyphus* sp. |  | Ghana | [89] |
|  | Macronyssidae | *Chiroptonyssus robustipes* |  | Ghana | [89] |
|  | Macronyssidae | *Liponyssus glutinosus* |  | Egypt | [70] |
|  | Macronyssidae | *Liponyssus longimanus* |  | Egypt | [70] |
|  | Macronyssidae | *Steatonyssus longipes* |  | Ghana | [89] |
|  | Pyemotidae | *Pyemotes* sp. |  | Ghana | [89] |
|  | Sarcoptidae | *Nycteridocoptes pteropodi* |  | Zaire^$^ | [99] |
|  | Sarcoptidae | *Nycteridocoptes rousetti* |  | Congo | [81, 100] |
|  | Sarcoptidae | *Teinocoptes eonycteri* |  | Egypt | [99] |
|  | Sarcoptidae | *Teinocoptes pahangensis* |  | Angola, Cyprus, Egypt | [99] |
|  | Sarcoptidae | *Teinocoptes rousetti* |  | Zaire | [99] |
|  | Spinturnicidae | *Ancystropus aethiopicus Hirst* |  |  | [101] |
|  | Spinturnicidae | *Ancystropus aequatorialis* |  | Equatorial Guinea | [102] |
|  | Spinturnicidae | *Ancystropus leleupi* |  | Congo | [81] |
|  | Spinturnicidae | *Ancystropus taprobanius* |  | Equatorial Guinea | [102] |
|  | Spinturnicidae | *Ancystropus zelebori* |  | Equatorial Guinea, South Africa, Egypt, Congo | [70, 81, 87, 102] |
|  | Spinturnicidae | *Ancystropus zeleborii Kolenati* |  | Egypt | [103] |
|  | Spinturnicidae | *Meristaspis kenyaensis* |  | Ghana, Egypt | [89, 103] |
|  | Spinturnicidae | *Meristaspis lateralis* |  | Iran, Egypt | [96, 103] |
|  | Spinturnicidae | *Periglischrus ojastii* |  | Ghana | [89] |
|  | Spinturnicidae | *Periglischrus paracutisternus* |  | Ghana | [89] |
|  | Spinturnicidae | *Spinturnix* sp. |  | Ghana | [89] |
|  | Spinturnicidae | *Spinturnix americana* |  | Ghana | [89] |
|  | Spinturnicidae | *Spinturnix delacruzi* |  | Equatorial Guinea | [102] |
|  | Spinturnicidae | *Spinturnix lateralis* |  | Republic of the Congo, Egypt, Palestine, Cyprus | [70, 81] |
| **Arthropod (ticks)** | Argasidae | *Alectorobius (Reticulinasus) camicasi* |  | Senegal^@^ | [70, 104] |
|  | Argasidae | *Argas boueti* |  | Egypt, Israel | [70] |
|  | Argasidae | *Argas vespertilionis* |  | Jordan, Israel, Sudan, Ghana | [70, 89, 90] |
|  | Argasidae | *Carios vespertilionis* |  | Western Palearctic | [105] |
|  | Argasidae | *Chiropterargas boueti* |  | Western Palearctic | [105] |
|  | Argasidae | *Ornithodoros (Reticulinasus) salahi* |  | Jordan*, Egypt, Israel, Palestine, Cyprus, Iran*, Oman "Western Palearctic" | [90] |
|  | Argasidae | *Ornithodoros faini* |  | South Africa | [87] |
|  | Ixodidae | *Ixodes* sp. |  | Ghana | [89] |

**References:**

1.	Rougeron V, Suquet E, Maganga GD, Jiolle D, Mombo IM, Bourgarel M, Motsch P, Arnathau C, Durand P, Drexler F, Drosten C, Renaud F, Prugnolle F, Leroy EM (2016) Characterization and phylogenetic analysis of new bat astroviruses detected in Gabon, Central Africa. Acta Virol 60:386–392

2.	Waruhiu C, Ommeh S, Obanda V, Agwanda B, Gakuya F, Ge XY, Yang XL, Wu LJ, Zohaib A, Hu B, Shi ZL (2017) Molecular detection of viruses in Kenyan bats and discovery of novel astroviruses, caliciviruses and rotaviruses. Virol Sin 32:101–114

3.	Tong S, Conrardy C, Ruone S, Kuzmin IV, Guo X, Tao Y, Niezgoda M, Haynes L, Agwanda B, Breiman RF, Anderson LJ, Rupprecht CE (2009) Detection of novel SARS-like and other coronaviruses in bats from Kenya. Emerg Infect Dis 15:482

4.	Li B, Si H-R, Zhu Y, Yang X-L, Anderson DE, Shi Z-L, Wang L-F, Zhou P (2020) Discovery of bat coronaviruses through surveillance and probe capture-based next-generation sequencing. Msphere 5:e00807–00819

5.	Anthony SJ, Johnson CK, Greig DJ, Kramer S, Che X, Wells H, Hicks AL, Joly DO, Wolfe ND, Daszak P, Karesh W, Lipkin WI, Morse SS; PREDICT Consortium; Mazet JAK, Goldstein T (2017) Global patterns in coronavirus diversity. Virus Evol 3:vex012

6.	Lacroix A, Vidal N, Keita AK, Thaurignac G, Esteban A, De Nys H, Diallo R, Toure A, Goumou S, Soumah AKLacroix A, Vidal N, Keita AK, Thaurignac G, Esteban A, De Nys H, Diallo R, Toure A, Goumou S, Soumah AK, Povogui M, Koivogui J, Monemou JL, Raulino R, Nkuba A, Foulongne V, Delaporte E, Ayouba A, Peeters M (2020) Wide diversity of coronaviruses in frugivorous and insectivorous bat species: a pilot study in Guinea, West Africa. Viruses 12:855

7.	Geldenhuys M, Ross N, Dietrich M, de Vries JL, Mortlock M, Epstein JH, Weyer J, Pawęska JT, Markotter W (2023) Viral maintenance and excretion dynamics of coronaviruses within an Egyptian rousette fruit bat maternal colony: considerations for spillover. Sci Rep 13:15829

8.	Pourrut X, Souris M, Towner JS, Rollin PE, Nichol ST, Gonzalez J-P, Leroy E (2009) Large serological survey showing cocirculation of Ebola and Marburg viruses in Gabonese bat populations, and a high seroprevalence of both viruses in *Rousettus aegyptiacus*. BMC Infect Dis 9:159

9.	Towner JS, Amman BR, Sealy TK, Carroll SA, Comer JA, Kemp A, Swanepoel R, Paddock CD, Balinandi S, Khristova ML, Formenty PB, Albarino CG, Miller DM, Reed ZD, Kayiwa JT, Mills JN, Cannon DL, Greer PW, Byaruhanga E, Farnon EC, Atimnedi P, Okware S, Katongole-Mbidde E, Downing R, Tappero JW, Zaki SR, Ksiazek TG, Nichol ST, Rollin PE (2009) Isolation of genetically diverse Marburg viruses from Egyptian fruit bats. PLoS Pathog 5:e1000536

10.	Pawęska JT, Storm N, Markotter W, Di Paola N, Wiley MR, Palacios G, van Vuren PJ (2020) Shedding of Marburg virus in naturally infected Egyptian rousette bats, South Africa, 2017. Emerg Infect Dis 26:3051

11.	Amman BR, Carroll SA, Reed ZD, Sealy TK, Balinandi S, Swanepoel R, Kemp A, Erickson BR, Comer JA, Campbell S, Cannon DL, Khristova ML, Atimnedi P, Paddock CD, Crockett RJ, Flietstra TD, Warfield KL, Unfer R, Katongole-Mbidde E, Downing R, Tappero JW, Zaki SR, Rollin PE, Ksiazek TG, Nichol ST, Towner JS (2012) Seasonal pulses of Marburg virus circulation in juvenile *Rousettus aegyptiacus* bats coincide with periods of increased risk of human infection. PLoS Pathog 8:e1002877

12.	Amman BR, Bird BH, Bakarr IA, Bangura J, Schuh AJ, Johnny J, Sealy TK, Conteh I, Koroma AH, Foday I, Amara E, Bangura AA, Gbakima AA, Tremeau-Bravard A, Belaganahalli M, Dhanota J, Chow A, Ontiveros V, Gibson A, Turay J, Patel K, Graziano J, Bangura C, Kamanda ES, Osborne A, Saidu E, Musa J, Bangura D, Williams SMT, Wadsworth R, Turay M, Edwin L, Mereweather-Thompson V, Kargbo D, Bairoh FV, Kanu M, Robert W, Lungai V, Guetiya Wadoum RE, Coomber M, Kanu O, Jambai A, Kamara SM, Taboy CH, Singh T, Mazet JAK, Nichol ST, Goldstein T, Towner JS, Lebbie A (2020) Isolation of Angola-like Marburg virus from Egyptian rousette bats from West Africa. Nat Commun 11:510

13.	Amman BR, Nyakarahuka L, McElroy AK, Dodd KA, Sealy TK, Schuh AJ, Shoemaker TR, Balinandi S, Atimnedi P, Kaboyo W, Nichol ST, Towner JS (2014) Marburgvirus resurgence in Kitaka Mine bat population after extermination attempts, Uganda. Emerg Infect Dis 20:1761

14.	Maganga GD, Bourgarel M, Ebang Ella G, Drexler JF, Gonzalez J-p, Drosten C, Leroy EM (2011) Is Marburg virus enzootic in Gabon? J Infect Dis 204:S800–S803

15.	Towner JS, Pourrut X, Albariño CG, Nkogue CN, Bird BH, Grard G, Ksiazek TG, Gonzalez J-P, Nichol ST, Leroy EM (2007) Marburg virus infection detected in a common African bat. PLoS One 2:e764

16.	Kuzmin IV, Niezgoda M, Franka R, Agwanda B, Markotter W, Breiman RF, Shieh W-J, Zaki SR, Rupprecht CE (2010) Marburg virus in fruit bat, Kenya. Emerg Infect Dis 16:352

17.	Kajihara M, Hang'ombe BM, Changula K, Harima H, Isono M, Okuya K, Yoshida R, Mori-Kajihara A, Eto Y, Orba Y, Ogawa H, Qiu Y, Sawa H, Simulundu E, Mwizabi D, Munyeme M, Squarre D, Mukonka V, Mweene A, Takada A (2019) Marburgvirus in Egyptian fruit bats, Zambia. Emerg Infect Dis 25:1577

18.	Kading RC, Schountz T (2016) Flavivirus infections of bats: potential role in Zika virus ecology. Am J Trop Med Hyg 95:993

19.	Simpson D, Williams M, O'Sullivan J, Cunningham J, Mutere F (1968) Studies on arboviruses and bats (Chiroptera) in East Africa: II.—Isolation and haemagglutination-inhibition studies on bats collected in Kenya and throughout Uganda. Ann Trop Med Parasitol 62:432–440

20.	Akov Y, Goldwasser R (1966) Prevalence of antibodies to arbovirus in various animals in Israel. Bull World Health Organ 34:901–909

21.	Quan PL, Firth C, Conte JM, Williams SH, Zambrana-Torrelio CM, Anthony SJ, Ellison JA, Gilbert AT, Kuzmin IV, Niezgoda M, Osinubi MO, Recuenco S, Markotter W, Breiman RF, Kalemba L, Malekani J, Lindblade KA, Rostal MK, Ojeda-Flores R, Suzan G, Davis LB, Blau DM, Ogunkoya AB, Alvarez Castillo DA, Moran D, Ngam S, Akaibe D, Agwanda B, Briese T, Epstein JH, Daszak P, Rupprecht CE, Holmes EC, Lipkin WI (2013) Bats are a major natural reservoir for hepaciviruses and pegiviruses. Proc Natl Acad Sci USA 110:8194–8199

22.	Simpson D, O’Sullivan J (1968) Studies on arboviruses and bats (Chiroptera) in East Africa: I.—Experimental infection of bats and virus transmission attempts in *Aedes* (*Stegomyia*) *aegypti* (Linnaeus). Ann Trop Med Parasitol 62:422–431

23.	Fagre AC, Lewis J, Miller MR, Mossel EC, Lutwama JJ, Nyakarahuka L, Nakayiki T, Kityo R, Nalikka B, Towner JS, Amman BR, Sealy TK, Foy B, Schountz T, Anderson J, Kading RC (2021) Subgenomic flavivirus RNA (sfRNA) associated with Asian lineage Zika virus identified in three species of Ugandan bats (family Pteropodidae). Sci Rep 11:8370

24.	Kalunda M, Mukwaya L, Mukuye A, Lule M, Sekyalo E, Wright J, Casals J (1986) Kasokero virus: a new human pathogen from bats (*Rousettus aegyptiacus*) in Uganda. Am J Trop Med Hyg 35:387–392

25.	Walker PJ, Widen SG, Firth C, Blasdell KR, Wood TG, Da Rosa APT, Guzman H, Tesh RB, Vasilakis N (2015) Genomic characterization of Yogue, Kasokero, Issyk-Kul, Keterah, Gossas, and Thiafora viruses: nairoviruses naturally infecting bats, shrews, and ticks. Am J Trop Med Hyg 93:1041

26.	Müller MA, Devignot S, Lattwein E, Corman VM, Maganga GD, Gloza-Rausch F, Binger T, Vallo P, Emmerich P, Cottontail VM, Tschapka M, Oppong S, Drexler JF, Weber F, Leroy EM, Drosten C (2016) Evidence for widespread infection of African bats with Crimean-Congo hemorrhagic fever-like viruses. Sci Rep 6:26637

27.	Kandeil A, Gomaa MR, Shehata MM, El Taweel AN, Mahmoud SH, Bagato O, Moatasim Y, Kutkat O, Kayed AS, Dawson P, Qiu X, Bahl J, Webby RJ, Karesh WB, Kayali G, Ali MA (2019) Isolation and characterization of a distinct influenza A virus from Egyptian bats. J Virol 93:10.1128/jvi. 01059–01018

28.	Harima H, Sasaki M, Kajihara M, Mori-Kajihara A, Hang'ombe BM, Changula K, Orba Y, Ogawa H, Simuunza M, Yoshida R, Mweene A, Takada A, Sawa H (2020) Detection of novel orthoreovirus genomes in shrew (*Crocidura hirta*) and fruit bat (*Rousettus aegyptiacus*). J Vet Med Sci 82:162–167

29.	Harima H, Sasaki M, Orba Y, Okuya K, Qiu Y, Wastika CE, Changula K, Kajihara M, Simulundu E, Yamaguchi T, Eto Y, Mori-Kajihara A, Sato A, Taniguchi S, Takada A, Saijo M, Hang'ombe BM, Sawa H (2021) Attenuated infection by a Pteropine orthoreovirus isolated from an Egyptian fruit bat in Zambia. PLoS Negl Trop Dis 15:e0009768

30.	Amponsah-Mensah K (2017) Ecology of Fruit Bats in Ghana, with Special Focus on *Epomophorus Gambianus*, and the Role of Fruit Bats in Zoonotic Disease Transmission. Digital Collections: University of Ghana

31.	Amman BR, Albariño CG, Bird BH, Nyakarahuka L, Sealy TK, Balinandi S, Schuh AJ, Campbell SM, Ströher U, Jones ME, Vodzack ME, Reeder DM, Kaboyo W, Nichol ST, Towner JS (2015) A recently discovered pathogenic paramyxovirus, Sosuga virus, is present in *Rousettus aegyptiacus* fruit bats at multiple locations in Uganda. J Wildl Dis 51:774–779

32.	Amman BR, Koroma AH, Schuh AJ, Conteh I, Sealy TK, Foday I, Johnny J, Bakarr IA, Whitmer SLM, Wright EA, Gbakima AA, Graziano J, Bangura C, Kamanda E, Osborne A, Saidu E, Musa JA, Bangura DF, Williams SMT, Fefegula GM, Sumaila C, Jabaty J, James FH, Jambai A, Garnett K, Kamara TF, Towner JS, Lebbie A (2024) Sosuga Virus Detected in Egyptian Rousette Bats (*Rousettus aegyptiacus*) in Sierra Leone. Viruses 16:648

33.	Mortlock M, Dietrich M, Weyer J, Pawęska JT, Markotter W (2019) Co-circulation and excretion dynamics of diverse rubula-and related viruses in Egyptian rousette bats from South Africa. *Viruses* 11:37

34.	Drexler JF, Corman VM, Müller MA, Maganga GD, Vallo P, Binger T, Gloza-Rausch F, Cottontail VM, Rasche A, Yordanov S, Seebens A, Knörnschild M, Oppong S, Adu Sarkodie Y, Pongombo C, Lukashev AN, Schmidt-Chanasit J, Stöcker A, Carneiro AJ, Erbar S, Maisner A, Fronhoffs F, Buettner R, Kalko EK, Kruppa T, Franke CR, Kallies R, Yandoko ER, Herrler G, Reusken C, Hassanin A, Krüger DH, Matthee S, Ulrich RG, Leroy EM, Drosten C (2012) Bats host major mammalian paramyxoviruses. Nat Commun 3:796

35.	Kading RC, Kityo RM, Mossel EC, Borland EM, Nakayiki T, Nalikka B, Nyakarahuka L, Ledermann JP, Panella NA, Gilbert AT, Crabtree MB, Peterhans JK, Towner JS, Amman BR, Sealy TK, Nichol ST, Powers AM, Lutwama JJ, Miller BR (2018) Neutralizing antibodies against flaviviruses, Babanki virus, and Rift Valley fever virus in Ugandan bats. Infect Ecol Epidemiol 8:1439215

36.	Picard‐Meyer E, Barrat J, Tissot E, Barrat M, Bruyere V, Cliquet F (2004) Genetic analysis of European bat lyssavirus type 1 isolates from France. Vet Rec 154:589–595

37.	Vora NM, Osinubi MOV, Davis L, Abdurrahman M, Adedire EB, Akpan H, Aman-Oloniyo AF, Audu SW, Blau D, Dankoli RS, Ehimiyein AM, Ellison JA, Gbadegesin YH, Greenberg L, Haberling D, Hutson C, Idris JM, Kia GSN, Lawal M, Matthias SY, Mshelbwala PP, Niezgoda M, Ogunkoya AB, Ogunniyi AO, Okara GC, Olugasa BO, Ossai OP, Oyemakinde A, Person MK, Rupprecht CE, et al (2020) Bat and lyssavirus exposure among humans in area that celebrates bat festival, Nigeria, 2010 and 2013. Emerg Infect Dis 26:1399

38.	Kuzmin IV, Niezgoda M, Franka R, Agwanda B, Markotter W, Beagley JC, Urazova OY, Breiman RF, Rupprecht CE (2008) Lagos bat virus in Kenya. J Clin Microbiol 46:1451–1461

39.	Kuzmin IV, Turmelle AS, Agwanda B, Markotter W, Niezgoda M, Breiman RF, Rupprecht CE (2011) Commerson's leaf-nosed bat (*Hipposideros commersoni*) is the likely reservoir of Shimoni bat virus. Vector Borne Zoonotic Dis 11:1465–1470

40.	Rønsholt L, Sørensen K, Bruschke C, Wellenberg G, Van Oirschot J, Johnstone P, Whitby J, Bourhy H (1998) Clinically silent rabies infection in (zoo) bats. Vet Rec 142:519–520

41.	Van der Poel W, Van der Heide R, Van Amerongen G, Van Keulen L, Wellenberg G, Bourhy H, Schaftenaar W, Groen J, Osterhaus A (2000) Characterisation of a recently isolated lyssavirus in frugivorous zoo bats. Arch Virol 145:1919–1931

42.	Wellenberg G, Audry L, Rønsholt L, Van der Poel W, Bruschke C, Bourhy H (2002) Presence of European bat lyssavirus RNAs in apparently healthy *Rousettus aegyptiacus* bats. Arch Virol 147:349–361

43.	Calisher CH, Childs JE, Field HE, Holmes KV, Schountz T (2006) Bats: important reservoir hosts of emerging viruses. Clin Microbiol Rev 19:531–545

44.	Sasaki M, Kajihara M, Changula K, Mori-Kajihara A, Ogawa H, Hang'ombe BM, Mweene AS, Simuunza M, Yoshida R, Carr M, Orba Y, Takada A, Sawa H (2018) Identification of group A rotaviruses from Zambian fruit bats provides evidence for long-distance dispersal events in Africa. Infect Genet Evol 63:104–109

45.	Fagre AC, Lee JS, Kityo RM, Bergren NA, Mossel EC, Nakayiki T, Nalikka B, Nyakarahuka L, Gilbert AT, Peterhans JK, Crabtree MB, Towner JS, Amman BR, Sealy TK, Schuh AJ, Nichol ST, Lutwama JJ, Miller BR, Kading RC (2019) Discovery and characterization of Bukakata orbivirus (Reoviridae: Orbivirus), a novel virus from a Ugandan bat. Viruses 11:209

46.	Fagre AC, Kading RC (2019) Can bats serve as reservoirs for arboviruses? Viruses 11:215

47.	Jansen van Vuren P, Allam M, Wiley MR, Ismail A, Storm N, Birkhead M, Markotter W, Palacios G, Pawęska JT (2018) A novel adenovirus isolated from the Egyptian fruit bat in South Africa is closely related to recent isolates from China. Sci Rep 8:9584

48.	Dietrich M, Kearney T, Seamark EC, Pawęska JT, Markotter W (2018) Synchronized shift of oral, faecal and urinary microbiotas in bats and natural infection dynamics during seasonal reproduction. R Soc Open Sci 5:180041

49.	Jánoska M, Vidovszky M, Molnár V, Liptovszky M, Harrach B, Benkő M (2011) Novel adenoviruses and herpesviruses detected in bats. Vet J 189:118–121

50.	Harima H, Qiu Y, Yamagishi J, Kajihara M, Changula K, Okuya K, Isono M, Yamaguchi T, Ogawa H, Nao N, Sasaki M, Simulundu E, Mweene AS, Sawa H, Ishihara K, Hang'ombe BM, Takada A (2023) Surveillance, Isolation, and Genetic Characterization of Bat Herpesviruses in Zambia. Viruses 15:1369

51.	Pozo F, Juste J, Vázquez-Morón S, Aznar-López C, Ibáñez C, Garin I, Aihartza J, Casas I, Tenorio A, Echevarría JE (2016) Identification of novel betaherpesviruses in Iberian bats reveals parallel evolution. PLoS One 11:e0169153

52.	Rector A, Mostmans S, Van Doorslaer K, McKnight CA, Maes RK, Wise AG, Kiupel M, Van Ranst M (2006) Genetic characterization of the first chiropteran papillomavirus, isolated from a basosquamous carcinoma in an Egyptian fruit bat: the *Rousettus aegyptiacus* papillomavirus type 1. Vet Microbiol 117:267–275

53.	McKnight CA, Wise AG, Maes RK, Howe C, Rector A, Van Ranst M, Kiupel M (2006) Papillomavirus-associated basosquamous carcinoma in an Egyptian fruit bat (*Rousettus aegyptiacus*). J Zoo Wildl Med 37:193–196

54.	Carr M, Gonzalez G, Sasaki M, Ito K, Ishii A, Hang’ombe BM, Mweene AS, Orba Y, Sawa H (2017) Discovery of African bat polyomaviruses and infrequent recombination in the large T antigen in the Polyomaviridae. J Gen Virol 98:726–738

55.	Tao Y, Shi M, Conrardy C, Kuzmin IV, Recuenco S, Agwanda B, Alvarez DA, Ellison JA, Gilbert AT, Moran D, Niezgoda M, Lindblade KA, Holmes EC, Breiman RF, Rupprecht CE, Tong S (2013) Discovery of diverse polyomaviruses in bats and the evolutionary history of the Polyomaviridae. J Gen Virol 94:738

56.	David D, Davidson I, Berkowitz A, Karniely S, Edery N, Bumbarov V, Laskar O, Elazari‐Volcani R (2020) A novel poxvirus isolated from an Egyptian fruit bat in Israel. Vet Med Sci 6:587–590

57.	David D, Davidson I, Karniely S, Edery N, Rosenzweig A, Sol A (2021) Israeli *Rousettus aegyptiacus* Pox Virus (IsrRAPXV) Infection in Juvenile Egyptian Fruit Bat (*Rousettus aegyptiacus*): Clinical Findings and Molecular Detection. Viruses 13:407

58.	Bai Y, Osinubi MOV, Osikowicz L, McKee C, Vora NM, Rizzo MR, Recuenco S, Davis L, Niezgoda M, Ehimiyein AM, Kia GSN, Oyemakinde A, Adeniyi OS, Gbadegesin YH, Saliman OA, Ogunniyi A, Ogunkoya AB, Kosoy MY; Idanre Bat Festival Investigation Team (2018) Human exposure to novel *Bartonella* species from contact with fruit bats. Emerg Infect Dis 24:2317

59.	Qiu Y, Kajihara M, Nakao R, Mulenga E, Harima H, Hang'ombe BM, Eto Y, Changula K, Mwizabi D, Sawa H, Higashi H, Mweene A, Takada A, Simuunza M, Sugimoto C (2020) Isolation of *Candidatus* Bartonella rousetti and other bat-associated Bartonellae from bats and their flies in Zambia. Pathogens 9:469

60.	Dietrich M, Tjale MA, Weyer J, Kearney T, Seamark EC, Nel LH, Monadjem A, Markotter W (2016) Diversity of *Bartonella* and *Rickettsia* spp. in bats and their blood-feeding ectoparasites from South Africa and Swaziland. PLoS One 11:e0152077

61.	Kosoy M, Bai Y, Lynch T, Kuzmin IV, Niezgoda M, Franka R, Agwanda B, Breiman RF, Rupprecht CE (2010) Bartonella spp. in bats, Kenya. Emerg Infect Dis 16:1875

62.	Stuckey MJ, Chomel BB, de Fleurieu EC, Aguilar-Setién A, Boulouis H-J, Chang C-C (2017) Bartonella, bats and bugs: a review. Comp Immunol Microbiol Infect Dis 55:20–29

63.	Modesto M, Satti M, Watanabe K, Puglisi E, Morelli L, Huang CH, Liou JS, Miyashita M, Tamura T, Saito S, Mori K, Huang L, Sciavilla P, Sandri C, Spiezio C, Vitali F, Cavalieri D, Perpetuini G, Tofalo R, Bonetti A, Arita M, Mattarelli P (2019) Characterization of *Bifidobacterium* species in feaces of the Egyptian fruit bat: Description of *B. vespertilionis* sp. nov. and *B. rousetti* sp. nov. Syst Appl Microbiol 42:126017

64.	Qiu Y, Nakao R, Hang'ombe BM, Sato K, Kajihara M, Kanchela S, Changula K, Eto Y, Ndebe J, Sasaki M, Thu MJ, Takada A, Sawa H, Sugimoto C, Kawabata H (2019) Human borreliosis caused by a new world relapsing fever borrelia–like organism in the old world. Clin Infect Dis 69:107–112

65.	Nowak K, Fahr J, Weber N, Lübke-Becker A, Semmler T, Weiss S, Mombouli JV, Wieler LH, Guenther S, Leendertz FH, Ewers C (2017) Highly diverse and antimicrobial susceptible *Escherichia coli* display a naïve bacterial population in fruit bats from the Republic of Congo. PLoS One 12:e0178146

66.	Han JE, Gomez DK, Kim JH, Choresca Jr CH, Shin SP, Park SC (2010) Isolation of a zoonotic pathogen *Kluyvera ascorbata* from Egyptian fruit-bat *Rousettus aegyptiacus*. J Vet Med Sci 72:85–87

67.	Dietrich M, Gomard Y, Lagadec E, Ramasindrazana B, Le Minter G, Guernier V, Benlali A, Rocamora G, Markotter W, Goodman SM, Dellagi K, Tortosa P (2018) Biogeography of *Leptospira* in wild animal communities inhabiting the insular ecosystem of the western Indian Ocean islands and neighboring Africa. Emerg Microbes Infect 7:1–12

68.	Di Cataldo S, Kamani J, Cevidanes A, Msheliza EG, Millán J (2020) Hemotropic mycoplasmas in bats captured near human settlements in Nigeria. Comp Immunol Microbiol Infect Dis 70:101448

69.	Held J, Gmeiner M, Mordmüller B, Matsiégui P-B, Schaer J, Eckerle I, Weber N, Matuschewski K, Bletz S, Schaumburg F (2017) Bats are rare reservoirs of *Staphylococcus aureus* complex in Gabon. Infect Genet Evol 47:118–120

70.	Weinberg M, Nissan Y, Yovel Y (2023) Egyptian Fruit Bat *Rousettus aegyptiacus* (E. Geoffroy, 1810). In: Chiroptera Handbook of the Mammals of Europe. Russo D, ed. Springer, Cham: 763–788

71.	Childs-Sanford SE, Kollias GV, Abou-Madi N, McDonough PL, Garner MM, Mohammed HO (2009) *Yersinia pseudotuberculosis* in a closed colony of Egyptian fruit bats (*Rousettus aegyptiacus)*. J Zoo Wildl Med 40:8–14

72.	Nakamura S, Settai S, Hayashidani H, Urabe T, Namai S, Une Y (2013) Outbreak of yersiniosis in Egyptian rousette bats (*Rousettus aegyptiacus*) caused by *Yersinia pseudotuberculosis* serotype 4b. J Comp Pathol 148:410–413

73.	Tocidlowski ME. (2003) Histoplasmosis outbreak at the Houston zoo. In: Annual Conference of the American Association of Zoo Veterinarians: 2003; Minneapolis, MN; 141–142

74.	Farina LL, Lankton JS (2018) Chiroptera In: Pathology of Wildlife and Zoo Animals. Elsevier: 607–633

75.	Akbar H, Pinçon C, Aliouat-Denis CM, Derouiche S, Taylor ML, Pottier M, Carreto-Binaghi LH, González-González AE, Courpon A, Barriel V, Guillot J, Chabé M, Suarez-Alvarez RO, Aliouat el M, Dei-Cas E, Demanche C (2012) Characterizing *Pneumocystis* in the lungs of bats: understanding *Pneumocystis* evolution and the spread of *Pneumocystis* organisms in mammal populations. Appl Environ Microbiol 78:8122–8136

76.	Childs-Sanford S, Garner M, Raymond J, Didier E, Kollias G (2006) Disseminated microsporidiosis due to *Encephalitozoon hellem* in an Egyptian fruit bat (*Rousettus aegyptiacus*). J Comp Pathol 134:370–373

77.	Fayed H, Shazly M, El-Monem S (2011) Life cycle of Eimeria rousetti sp. nov. (Alveolata: Apicomplexa: Eimeriidae) infecting the frugivorous bat, *Rousettus aegyptiacus* Geoffroy, 1810 (Mammalia: Chiroptera: Pteropodidae) in Egypt. J Am Sci 7:292–305

78.	Abd El Maleck BS, Abed GH, Maze N, Khalifa R (2015) Morphological and Ultrastructural of a New Species from Cephaline Gregarinidae Infected Fruit Egyptian Bat (*Rousettus aegyptiacus*) and its Vector. J Bacteriol Parasitol 6:1

79.	Atama N, Manu S, Ivande S, Rosskopf SP, Matuschewski K, Schaer J (2019) Survey of *Hepatocystis* parasites of fruit bats in the Amurum forest reserve, Nigeria, identifies first host record for *Rousettus aegyptiacus*. Parasitology 146:1550–1554

80.	Adam J-P (1965) Transmission d'hemosporidies par des anophèles cavernicoles dans les grottes du Congo (Brazzaville). Bull World Health Organ 32:598–602

81.	de Faveaux MA (1965) Les Parasites des Chiroptères-Rôle épidémiologique chez les Animaux et l’Homme au Katanga. Ann Parasitol Hum Comp 40:21–38

82.	Witsenburg F. (2014) The role of bat flies (Nycteribiidae) in the ecology and evolution of the blood parasite *Polychromophilus* (Apicomplexa: Haemosporida). Université de Lausanne, Faculté de Biologie et Médecine

83.	Clément L, Dietrich M, Markotter W, Fasel NJ, Monadjem A, López-Baucells A, Scaravelli D, Théou P, Pigeault R, Ruedi M, Christe P (2020) Out of Africa: the origins of the protozoan blood parasites of the *Trypanosoma cruzi* clade found in bats from Africa. Mol Phylogenet Evol 145:106705

84.	Stevens J, Teixeira M, Bingle L, Gibson W (1999) The taxonomic position and evolutionary relationships of *Trypanosoma rangeli*. Int J Parasitol 29:749–757

85.	Abed G, Khalifa R, A Mazen N, Abd-Elmaleck B (2005) Light and electron microscopic studies on the microfilariae of *Diptalonema vitae* Derpkagorskaya, 1923 (Nematoda: Filariidae) parasitizing the blood of fruit bats *Rousettus aegyptiacus aegyptiacus* at Assiut locality. Assiut Vet Med J 51:1–5

86.	Reinhardt K, Naylor RA, Siva-Jothy MT (2008) Temperature and humidity differences between roosts of the fruit bat, *Rousettus aegyptiacus* (Geoffroy, 1810), and the refugia of its ectoparasite, *Afrocimex constrictus*. Acta Chiropt 10:173–176

87.	Braack L (1989) Arthropod inhabitants of a tropical cave ‘island’ environment provisioned by bats. Biol Conserv 48:77–84

88.	Benda P, Abi Said MR, Bou Jaoude I, Karanouh R, Lučan RK, Sadek R, Ševčík M, Uhrin M, Horáček I (2016) Bats (Mammalia: Chiroptera) of the Eastern Mediterranean and Middle East. Part 13. Review of distribution and ectoparasites of bats in Lebanon. Acta Soc Zool Bohem 80:207–316

89.	Nartey N (2015) Common parasites of fruit-eating bats in southern Ghana. Accra (Ghana): University of Ghana 1–144

90.	Benda P, Lucan R, Obuch J, Reiter A, Andreas M, Backor P, Bohnenstengel T, Eid EK, Sevcik M, Vallo P, Amr ZS (2010) Bats (Mammalia: Chiroptera) of the Eastern Mediterranean and Middle East. Part 8. Bats of Jordan: fauna, ecology, echolocation, ectoparasites. Acta Soc Zool Bohem 74:185–353

91.	Špitalská E, Ševčík M, Peresh Y-Y, Benda P (2024) Bartonella in bat flies from the Egyptian fruit bat in the Middle East. Parasitol Res 123:144

92.	Benda P, Faizolâhi K, Andreas M, Obuch J, Reiter A, Ševčík M, Uhrin M, Vallo P, Ashrafi S (2012) Bats (Mammalia: Chiroptera) of the Eastern Mediterranean and Middle East. Part 10. Bat fauna of Iran. Acta Soc Zool Bohem 76:163–582

93.	Jansen van Vuren P, Wiley M, Palacios G, Storm N, McCulloch S, Markotter W, Birkhead M, Kemp A, Pawęska JT (2016) Isolation of a novel fusogenic orthoreovirus from *Eucampsipoda africana* bat flies in South Africa. Viruses 8:65

94.	Obame-Nkoghe J, Rahola N, Bourgarel M, Yangari P, Prugnolle F, Maganga GD, Leroy E-M, Fontenille D, Ayala D, Paupy C (2016) Bat flies (Diptera: Nycteribiidae and Streblidae) infesting cave-dwelling bats in Gabon: diversity, dynamics and potential role in Polychromophilus melanipherus transmission. Parasites & vectors 9:1–12

95.	Peirce M (1984) Parasites of Chiroptera in Zambia. J Wildl Dis 20:153–154

96.	Malek-Hosseini MJ, Sadeghi S, Bakhshi Y, Dashan M (2016) Ectoparasites (Insecta and Acari) associated with bats in south and south-western caves of Iran. Ambient Science 3:22–28

97.	Çetin H, Coşkun G, Dick CW (2020) Ectoparasitic Bat Flies (*Eucampsipoda hyrtlii*) Detected on the Egyptian Fruit Bat (*Rousettus aegyptiacus*) in Antalya, Turkey. Turkiye Parazitol Derg 44:115–117

98.	Maa T (1971) Review of the Streblidae (Diptera) parasitic on megachiropteran bats. Pacific Insects Monograph 28:213–243

99.	Klompen J (1992) Phylogenetic relationships in the mite family Sarcoptidae (Acari: Astigmata). Ann Arbor, Michigan: Museum of Zoology, The University of Michigan

100.	Fain A (1959) Les Acariens psoriques parasites des Chauves-souris VII.–Nouvelles observations sur le genre *Nycteridocoptes* Oudemans 1898. Acarologia 1:335–353

101.	Hafez S, Ebaid N, Tharwat M, Abdel-Megeed A (1994) Bat mites of Egypt (Acari: Spinturnicidae). Ann Agri Sci (Cairo) 39:463–471

102.	Estrada-Peña A, Ballesta RJ, Ibañez C (1992) Three new species of spinturnicid mites (Acari: Mesostigmata) from bats in Africa. Int J Acarol 18:307–313

103.	Negm MW, Fakeer MM (2014) Rediscovery of *Meristaspis lateralis* (Kolenati) (Acari: Mesostigmata: Spinturnicidae) parasitizing the Egyptian fruit bat, *Rousettus aegyptiacus* (Geoffroy) (Mammalia: Chiroptera), with a key to mites of bats in Egypt. J Egypt Soc Parasitol 44:25–32

104.	Sylla M, Cornet J, Marchand B (1997) Description of *Alectorobius* (*Reticulinasus*) *camicasi* sp. nov.(Acari: Ixodoidea: Argasidae), a parasite of fruit bats *Rousettus aegyptiacus* *occidentalis* in Senegal. Acarologia 38:239–254

105.	Sándor AD, Mihalca AD, Domşa C, Péter Á, Hornok S (2021) Argasid ticks of palearctic bats: distribution, host selection, and zoonotic importance. Front Vet Sci 8:684737
